# Advances in terahertz metasurface graphene for biosensing and application

**DOI:** 10.1186/s11671-023-03814-8

**Published:** 2023-04-15

**Authors:** Hao Bi, Maosheng Yang, Rui You

**Affiliations:** 1 Beijing Key Laboratory of Optoelectronic Measurement Technology, Beijing Information Science and Technology University, Beijing, China; 2 Beijing Laboratory of Biomedical Detection Technology and Instrument, Beijing Information Science and Technology University, Beijing, China; 3 Beijing Advanced Innovation Center for Integrated Circuits, 100084, Beijing, China; 4 School of Electrical and Optoelectronic Engineering, West Anhui University, Lu’an, 237012 China

**Keywords:** Graphene, Terahertz, Metasurfaces, Biosensing

## Abstract

Based on the extraordinary electromagnetic properties of terahertz waves, such as broadband, low energy, high permeability, and biometric fingerprint spectra, terahertz sensors show great application prospects in the biochemical field. However, the sensitivity of terahertz sensing technology is increasingly required by modern sensing demands. With the development of terahertz technology and functional materials, graphene-based terahertz metasurface sensors with the advantages of high sensitivity, fingerprint identification, nondestructive and anti-interference are gradually gaining attention. In addition to providing ideas for terahertz biosensors, these devices have attracted in-depth research and development by scientists. An overview of graphene-based terahertz metasurfaces and their applications in the detection of biochemical molecules is presented. This includes sensor mechanism research, graphene metasurface index evaluation, protein and nucleic acid sensors, and other chemical molecule sensing. A comparative analysis of graphene, nanomaterials, silicon, and metals to develop material-integrated metasurfaces. Furthermore, a brief summary of the main performance results of this class of devices is presented, along with suggestions for improvements to the existing shortcoming.

## Highlights

- Terahertz supersurfaces and graphene materials for spectroscopic analysis.
- Elucidating the mechanism of terahertz detection of biochemical molecules.
- Application of terahertz to biochemical molecule detection.

## Introduction

**Fig. 1:**
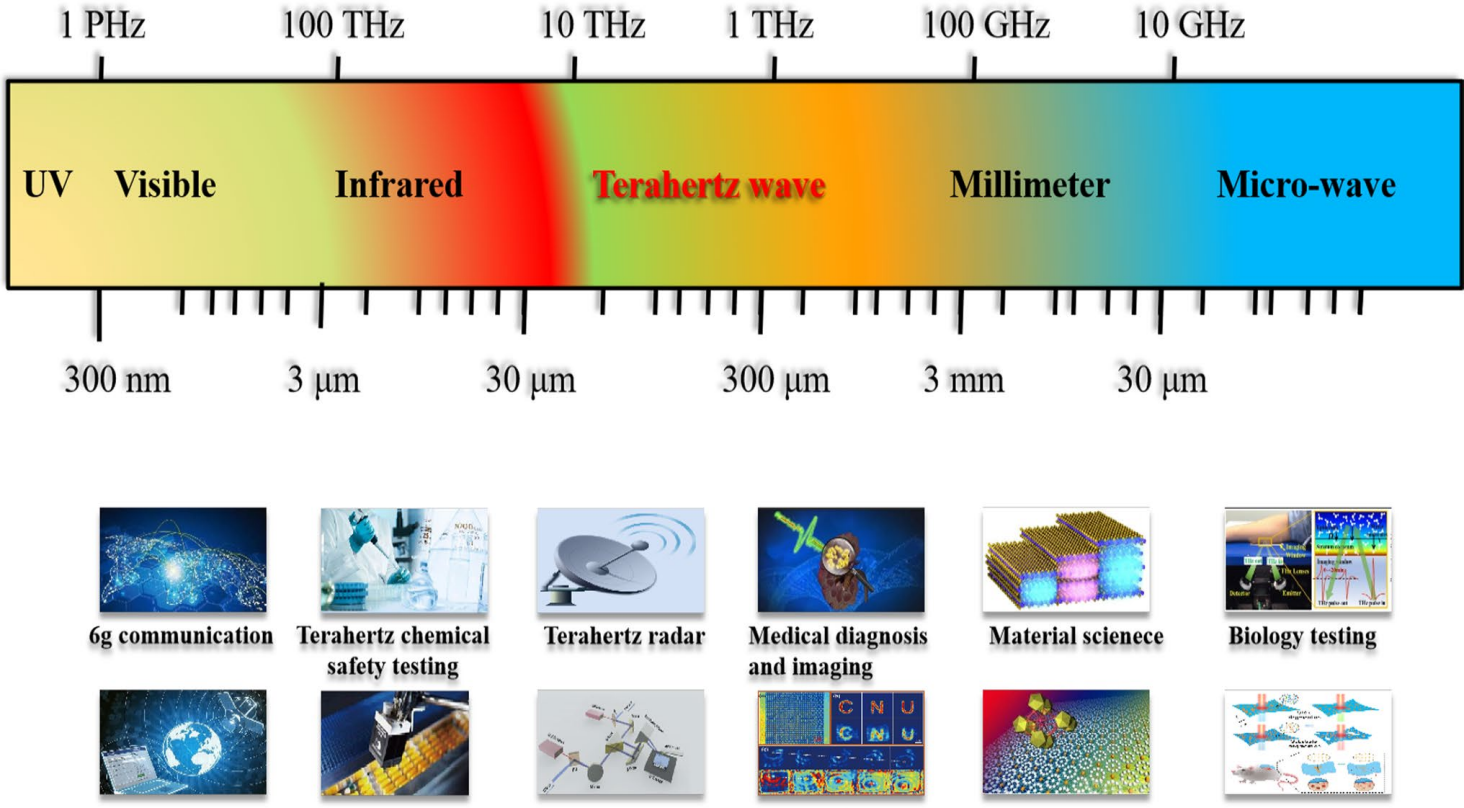
Schematic of the application of terahertz technology in related fields

Terahertz waves are electromagnetic waves with a wavelength of 0.03–3 mm and a frequency in the range of 0.1–10 THz [1]. Electromagnetic waves occupy a special place in modern optics and are a key field of spectroscopy (Fig. 1). It has electromagnetic wave characteristics such as broadband, low energy, and biomolecular fingerprinting [2]. Currently, many research groups are conducting fundamental research on terahertz metasurface sensing and its practical applications. Depending on the topology optimization of the metasurface structure, its sensitivity can be effectively enhanced, thereby expanding its potential applications.

**Fig. 2:**
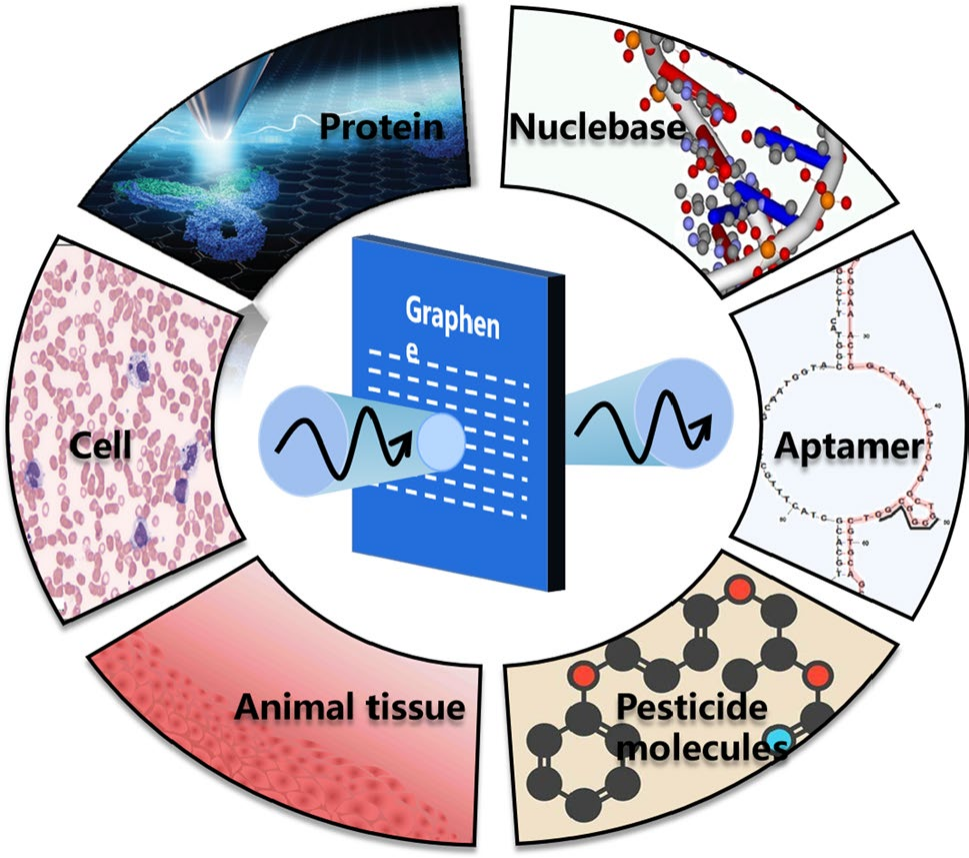
Schematic of biochemical application of terahertz spectroscopy

Terahertz waves lie between infrared rays and microwaves. The response of biochemical molecules to terahertz radiation mainly comes from collective vibrational modes, such as vibrations and conformational bending, determined by biochemical molecular conformation and chemical bonding. Biochemical molecules dominate intramolecular vibrations, including weak interactions, hydrogen bonds, van der Waals forces, etc. [3–6], providing the basis for the detection of biochemical molecules. Terahertz technology is widely used, including 6G communication [7], terahertz radar [8], imaging, biochemical detection, and medical diagnosis [9–11]. As shown in Fig. 2, using terahertz sensing to detect prohibited chemicals, aptamers, molecules, pesticides, proteins, cells, animal tissue, and other substances produces excellent results [5, 12]. However, terahertz sensing techniques do not apply to the detection of trace biochemical molecules. The corresponding bio-detection accuracy and detection frequency also need to be improved. In addition, the size of the biomolecular structure is smaller than the combined incident terahertz wavelength and the terahertz waves. Tiny perturbation features are difficult to resonate, which undoubtedly hinders the development of terahertz biosensors. To improve the detection sensitivity of the sensor, methods combining terahertz with a metasurface have been developed successively. Artificial materials with subwavelength unit geometrical properties consist of artificially designed periodic and nonperiodic arrangements with dimensions insignificant to the external stimulus wavelength. Metasurfaces have unique electromagnetic properties not found in natural materials [13, 14]. Metasurface sensing can effectively enhance the terahertz signal and has attracted attention in different fields.

**Fig. 3:**
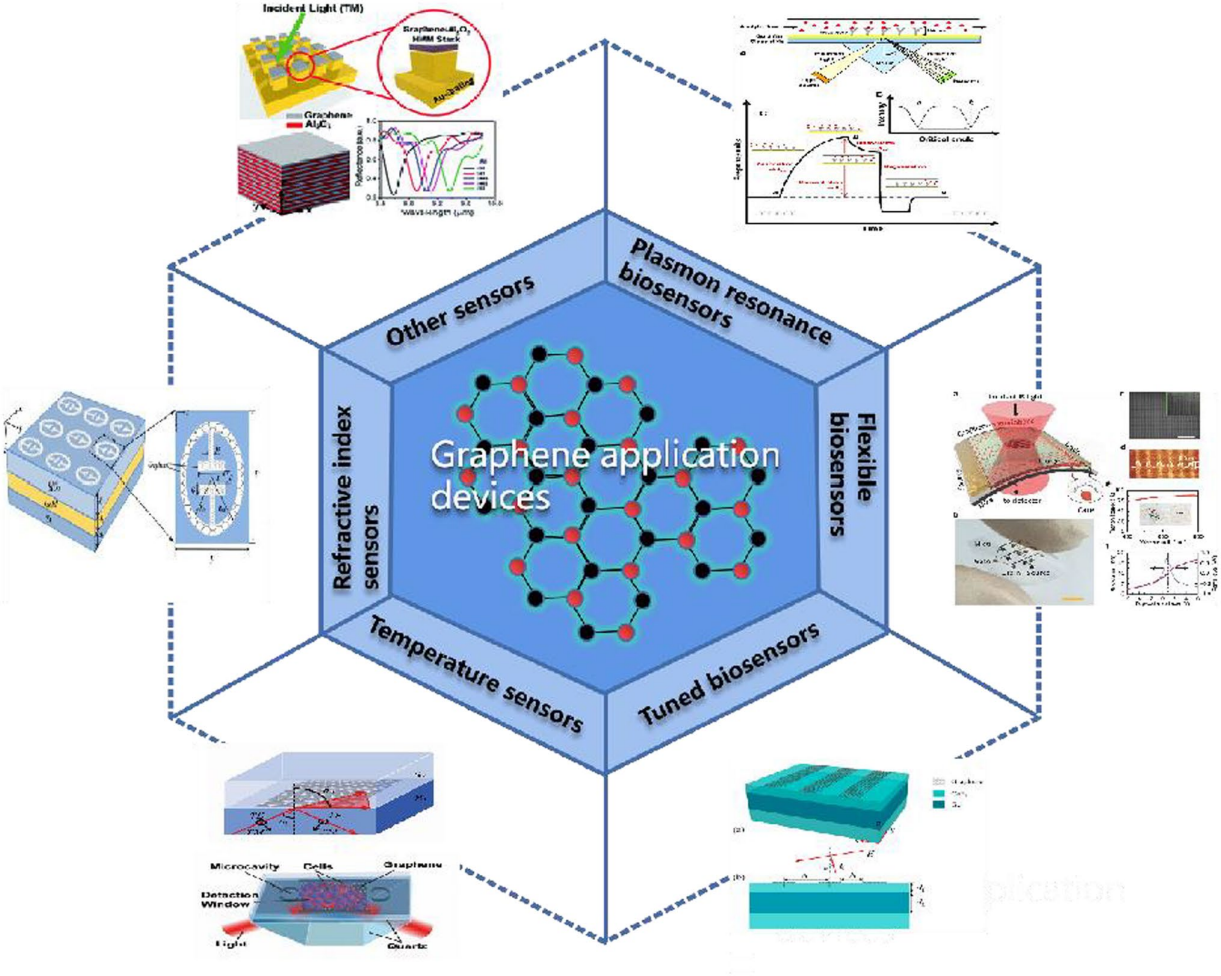
Graphene metamaterials for sensing applications

There is a need to find high-performance and stabilizing terahertz devices. Graphene as a two-dimensional material has been widely used in recent years [15]. Combining terahertz metasurfaces with graphene can provide a novel way to solve these problems because of its unique optoelectronic properties [16]. When designing high-performance terahertz sensor devices, the properties of the relevant material are crucial. Graphene materials with high electron transfer rates, high carrier mobility and low signal-to-noise ratio are essential for the highly sensitive detection of biological targets and other analytes in biological samples [17]. Graphene surfaces offer biological target active binding sites. By combining terahertz ultra-surfaces with graphene, the composite reflects comprehensive advantages [18]. The mutual coupling mechanism between terahertz metasurfaces and graphene is explored. Terahertz biosensors with metasurfaces have fingerprint spectra for biological macromolecules. The molecular vibrations of biological macromolecules have characteristic absorption peaks in the terahertz frequency range [19]. For biological small molecules, the terahertz wavelength is much longer than the size of small molecules and cannot be directly coupled with terahertz waves. However, the metasurfaces can be mutually coupled with terahertz waves, and by changing the structure of the metasurfaces, a local electric field can be generated, as shown in Fig. 3. The detection of biological small molecules can be achieved by using the change of electric field [20]. Graphene has various forms, such as graphene oxide (GO) [21], reduced graphene (RGO) [20], and graphene nanoribbons (GNRs) [22]. It has gained a lot of attention in the field of terahertz technology methods. Graphene has recently been combined with metasurfaces to form various biosensors for sensing parameters, such as temperature sensors, refractive index sensors, plasmon resonance biosensors, flexible sensors, and tuned biosensors.

The challenges faced by terahertz biomolecular sensors include metasurfaces that have the ability to be efficient and selective in capturing specific biomolecular target substances. To enhance the detection of biochemical molecules target substances on the metasurfaces improve the detection effect [23]. Among them, the two-dimensional nanomaterial graphene has been well developed and applied as a candidate material for biosensor coating on metasurfaces [24] and improves the possibility of biochemical binding target fixation, thus improving the field strength resonance effect signal strength; graphene is a two-dimensional material with only one carbon atom thickness; it is discovered by Andre Geim and Konstantin Novoselov, physicists at the University of Manchester, UK, who won the Nobel Prize in Physics in 2010 [25] for extracting and isolating graphene from graphite. It has extensive applications in materials science, micro- and nano-processing, energy, bio-detection and drug delivery and is considered a revolutionary material of the future. Graphene has a 0.142 nm carbon–carbon bond and is a very stable material with good mechanical competitiveness and photoelectronic properties [26, 27]. Combined with the excellent properties of graphene, it has not yet achieved industrial development, but it has potential applications as given in Table 1 [28–46]. In recent years, the development of metasurfaces unit structures and new materials has made the generation and detection of terahertz radiation a reality [47]. With the continuous exploration of terahertz theory, the foundation has been laid for the generation and detection of terahertz waves [48]. Compared with traditional biosensors, terahertz biosensors have many advantages, such as low cost, label-free detection, transitory time, and real-time detection.

**Table 1:** The potential applications of graphene

Application areas	Specific application
Medicine	Tissue engineering [28], Contrast medium [29], Biomedical sensors [30], Filtration of Biological samples [31]
Electronics	Optoelectronics [32], Photodetector [33], Filled conductive polymers [34]
Battery energy storage	Supercapacitors [35], Hydrogen storage [36], Batteries photoanode preparation [37]
Sensor	Sensor gas-sensitive [38], Infrared sensors [39], Contact lenses [40], Refractive index sensor [41], Temperature sensor [42], Magnetic sensors [43]
Other areas	Molecular sieve [44], Absorber [45], Modulation [46]

## Fundamental theoretical aspects in the terahertz metasurfaces field

In the detection of terahertz metasurfaces sensing, when terahertz waves pass through the metasurfaces unit structure and some periodic subwavelength resonators with small thickness are added to the medium, the distributions of the reflection and refraction field at the boundary of the medium determine the boundary of the homogeneous medium, two reflect and refraction properties [49]. Terahertz metasurfaces are biochemical sensors that induce strong resonance mechanisms derived from LC resonance, dipole resonance, and surface plasmon excitonic modes caused by displacement currents at the surface [19, 50–53]. As the periodic subwavelength resonators change the boundary conditions, the reflection and refraction coefficients are also transformed. The incident electromagnetic waves are coupled and propagated along the metasurfaces. And accompanied by charge oscillations within the resonator, these metasurfaces electromagnetic waves and oscillations for surface plasmonic excitation elements [54]. The local electronic field enhancement gives rise to the terahertz metasurfaces makes the detection of target biochemical molecules more sensitive. With the in-depth study of terahertz waves and the development of material science, metamaterial interfaces based on graphene have great potential for applying in the fields of terahertz biosensor design, molecular analysis was done and medical imaging [55, 56]. By using the periodic structure of the two-dimensional material graphene arranged as a unitary structure to interact with the incident electromagnetic waves, a resonant mode of structural neutron excitation on the surface is induced, which is made more sensitive to changes in the surrounding environment by the enhancement of the local electric field in the near field.

**Fig. 4:**
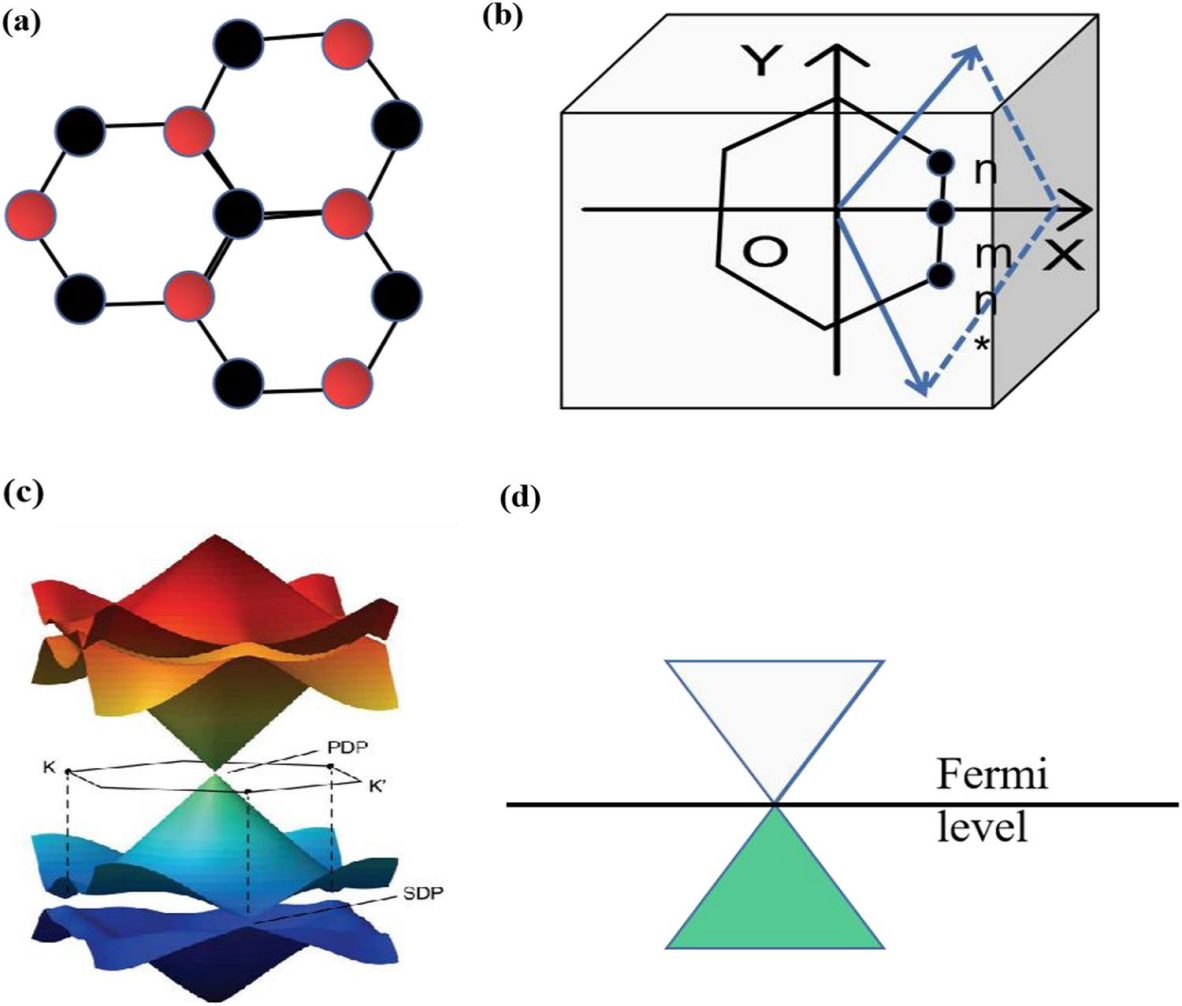
**a** Hexagonal lattice structure of graphene, **b** Brillouin zone, **c** intrinsic graphene band mechanism, and **d** free-stand graphene [58]. copyright 2017, Carbon

The four of valence electrons populate the sp orbitals, forming an extra-domain electron cloud, making graphene with ultra-high carrier migration, and resonance conditions of the electric field in order to excite the plasma resonance, graphite can be actively modulated by doping and applying gate pressure [57, 58], as shown in Fig. 4. The energy spectrum of the graphene energy band diagram of graphene at the Dirac point, where the movement of the fermi energy level at the Dirac point the resonance appears and disappears [59].

Surface plasmon resonance (SPR) is also a sensing mechanism, and sensors based on SPR through the interaction between the sensing element and the metasurfaces [60, 61]. This mechanism is through the target biochemical molecule binding, molecules concentration changes and chemical changes lead to changes in the local refractive index gradient, which excites the excitation of the surface plasma, generating terahertz waves through the frequency shift of the resonance peak. Terahertz waves metasurfaces coupled with graphene produce electromagnetic [62, 63]. Maxwell's set of equations can be calculated as shown in Eqs. (1)–(4).

1 $$\nabla \cdot D = \rho f$$

2 $$\nabla \cdot B = 0$$

3 $$\nabla \cdot E = - \frac{\partial B}{{\partial t}}$$

4 $$\nabla \cdot H = Jf + \frac{\partial D}{{\partial t}}$$

Considering the scattering of the graphene plasma on the time discontinuity for a confined plasma, the projection and reflection coefficients of the photons in the plasma are analyzed, as given in Eqs. (5)–(6). It considers rapidly varying time dispersion of the graphene plasma, by using the Laplace transform according to the Maxwell equations mentioned above to obtain the general Eqs. (7)–(8) for the time projection and reflection coefficients of the carriers. At low densities, by the above set of Maxwell equations can be obtained the electromagnetic corresponding generated by the terahertz coupling with the metasurface, according to the theoretical description of the classical electrodynamics [64–66]. The relationship between the macroscopic electric field and the magnetic field is described, and Maxwell's integral equations can be transformed into a linear system of equations solved to obtain the variation in the frequency domain.

5 $$\left| {t_{H} } \right| = \frac{{\left( {1 + \gamma } \right)}}{{\left( {2\gamma } \right)}}$$

6 $$\left| {r_{H} } \right| = \frac{{\left( {1 + \gamma } \right)}}{{\left( {2\gamma } \right)}}$$

7 $$D = \varepsilon_{0} E + P = \varepsilon E$$

8 $$H = \frac{1}{{u_{0} }}B - M = \frac{1}{u}B$$

Terahertz biochemical sensing developed by applying graphene involved in the design of the metasurfaces has enabled the detection of biochemical molecules such as; proteins, nucleic acids, viruses, pesticides, and other biochemical molecules. The electromagnetic environment in the vicinity of the graphene metasurfaces sensing can be significantly enhanced by the change in Fermi energy level caused by external induction, graphene metasurfaces to enhance sensor detection sensitivity.

## Terahertz metamaterial sensing detection index

Terahertz waves are associated with subwavelength resonant cell unit structures and material properties. Terahertz wave metasurfaces are combined with graphene, and their metasurfaces interact to trigger plasmon resonant modes, circulating dipole polarization, Fano resonances, and capacitive resonance [67, 68]. The researchers were able to rationally construct the metasurfaces and the resonances generated by the combination of materials.

Not only can the flexible manipulation of reflection, transmission and absorption resonances at arbitrary fixed frequency points be achieved, but also phase and frequency shift tuning of special properties. The performance indicators for capturing and detecting terahertz graphene metasurfaces mainly include quality factor (*Q*), sensitivity (*S*), and resonance characteristics (FOM) [69–72]. The quality factor *Q* reflects the quality of the resonance and reflects the resonance field enhancement produced by the metasurfaces resonance unit. The *Q* value is defined as (9).

9 $$Q = \frac{\Delta f}{{{\text{FWHM}}}}$$

$\Delta$*f* is the resonant center frequency and FWHM is the half-peak width.

When the biochemical molecules to be detected cause changes in the frequency of the resonance spectrum, it can be used for the purpose of qualitative and quantitative detection of the biochemical molecules to be tested measured, and real-time detection of targets. Sensitivity is an index to measure the change of resonant frequency of biochemical target molecules with the refractive index, and it is an important index for the detection performance of terahertz metasurface detection, which can be expressed as (10).

10 $$S = \frac{\Delta f}{{\Delta n}}$$

Δ*f* is the resonance shift and Δ*n* is the amount of variation in the molecular change properties of the target to be measured. Resonance characteristics FOM to compare the sensing performance of metamaterial sensors. FOM can be expressed as Eq. (11).

11 $${\text{FOM}} = S * Q$$

The FOM reveals the resonant properties of the sensor metasurface itself and the corresponding capabilities of the measured biochemical molecules. This equation can be understood as the relationship between the factors controlling the detection performance to balance the resonance characteristics of the sensor device and improve the interaction with the biochemical molecules to be detected. Research into terahertz sensor technology and detection is a top priority.

### Graphene terahertz metasurface sensing

Graphene has been identified as one of the most attractive materials in recent decades [73]. Graphene has been recognized as one of the most attractive materials in the last few decades. Due to its unique physical, chemical, electrical and mechanical properties, graphene shows great promise in many applications, including electronics, energy storage and sensor systems [74–76]. In particular, graphene-based terahertz sensor devices have been widely developed and are considered to be attractive platforms for fast and accurate detectors, fast and accurate sensing detectors. Graphene field effects can be used to detect individual atoms, biochemical molecules, gaseous chemicals, etc. [28, 77, 78]. Graphene has been applied to terahertz devices as an ideal biosensing material, which is inexpensive, environmentally friendly, and has a uniform arrangement of active sites containing a large number of functional groups such as hydroxyl and carboxyl groups, as well as excellent solubility properties that allow tight binding to biomolecules, and thereby terahertz metasurface for enhancing sensor sensitivity [79, 80]. Terahertz refractive index sensors are of tremendous interest due to their high sensitivity and good selectivity, and research has made substantial progress in this area in recent years. Through a synergistic interaction with metasurface, the continued development of graphene materials has improved the functionality and biocompatibility of terahertz biosensors.

**Fig. 5:**
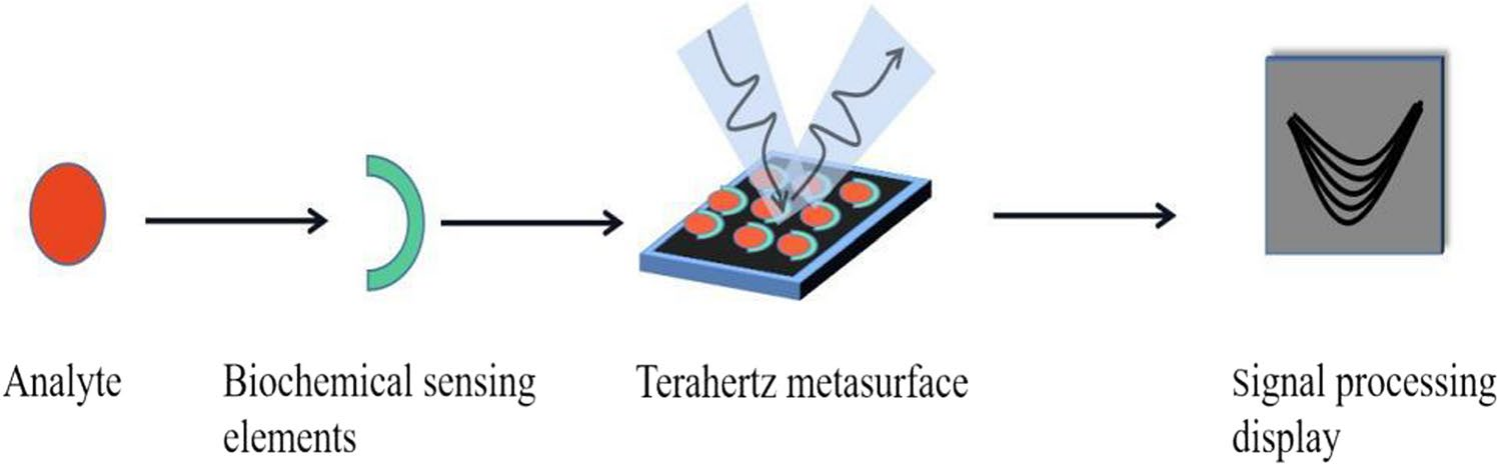
Schematic diagram of the principle of terahertz metasurface sensor

Graphene terahertz biosensor is a new detection and analysis method based on the development of the terahertz wave spectroscopy analysis method, as shown in Fig. 5; the graphene metasurface is used as the original component of signal conversion, and the target molecule is immobilized, the biochemical molecules are placed in the metasurface structure, and the specific recognition of effector between biochemical molecules, through the Fourier formula changer reaction of the signal converted to electrical signals, achieves the target of biochemical molecules qualitative and quantitative detection.

**Fig. 6:**
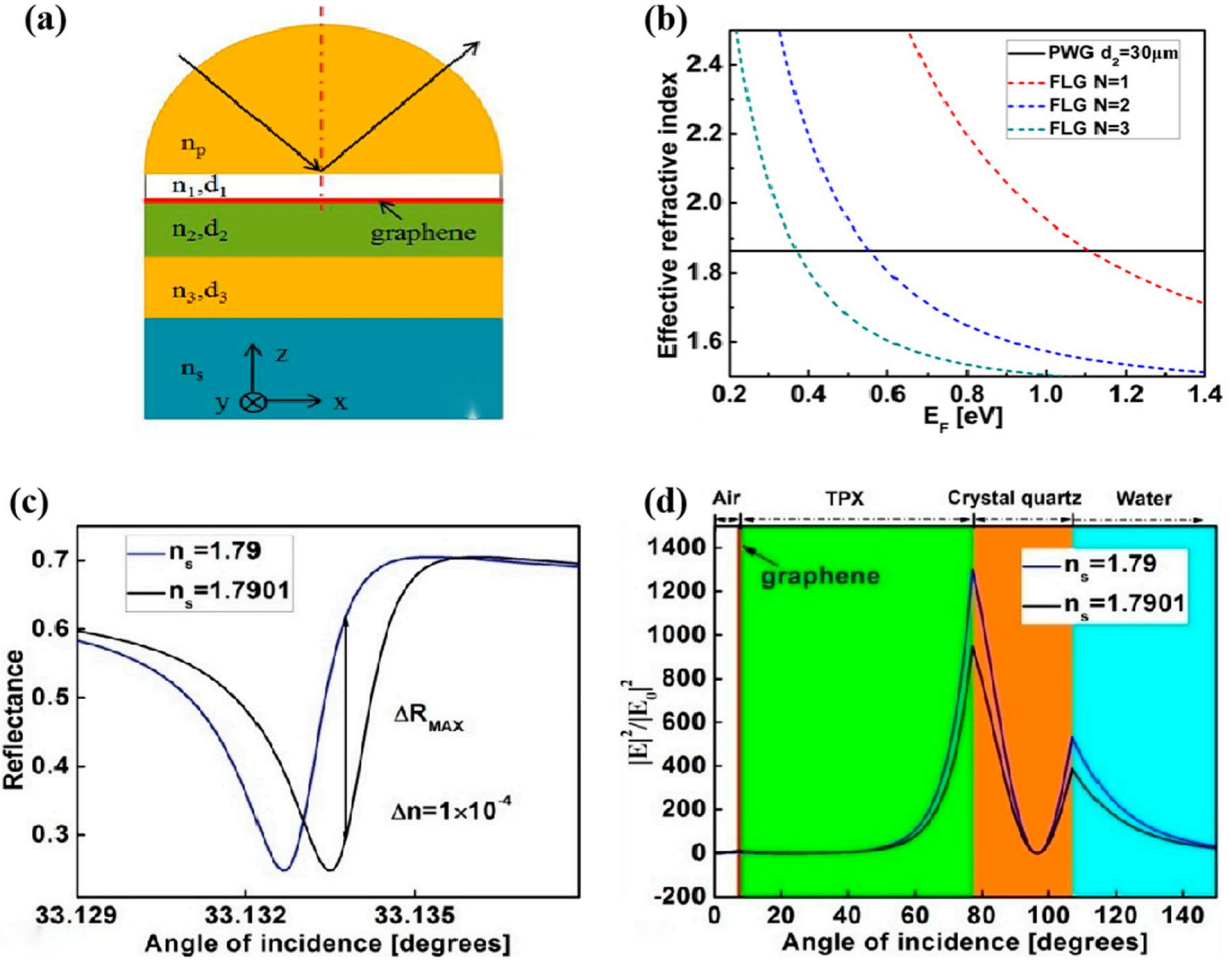
**a** Schematic diagram of the proposed biosensor based on Fano resonance, **b** Effective refractive indices of graphene SPP and PWG modes, **c** Shift of Fano line shape for the structure with *d*_1_ = 7 μm, *d*_2_ = 70 μm, *E*_*p*_ = 0.35 eV, **d** Schematic diagram of the electric field distributions for the proposed Fano resonance sensor at *θ* = 33.1332°. [81] copyright 2017, Sensor

Ruan et al. developed an ultrasensitive terahertz biosensor with a graphene, waveguide hybrid Fano structure resonance as shown in Fig. 6. Graphene plasmonic excitation offers promise for the development of functional devices in the terahertz band. This sensor was demonstrated by coupling graphene surface plasmon polarization and planar waveguide modes, and Fano resonance was achieved for the preparation of an ultrasensitive sensor [81]. The effects of the coupling layer and air layer thickness in the sandwich structure on the Fano resonance are also discussed in detail, and a sensitivity of 3260 RIU^−1^ is achieved. Meanwhile, Zhang et al. [82] proposed a graphene tunable terahertz sensor with dual Fano resonance and achieved ultrasensitive detection with 1.9082 THz per refractive index unit (RIU) and a quality factor (FOM) of 6.5662.

**Fig. 7:**
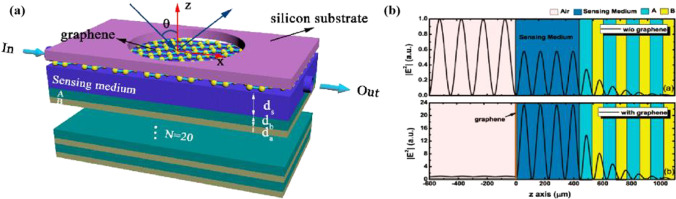
Schematic diagram of the proposed terahertz sensor **a** where the graphene is coated on the sensing medium and fastened by a fixing device, while a one-dimensional photonic crystal is beneath the sensing medium. **b** a) The normalized electric field distributions in the proposed structure in the absence of monolayer graphene. b) The normalized electric field distributions in the proposed structure coated with monolayer graphene. [83] Copyright 2020, Nanomaterials

Tang et al. [83] proposed a highly sensitive optical biochemical sensor at terahertz frequency, in which a sensing medium is sandwiched between a composite structure containing a one-dimensional photonic crystal and graphene, consisting of TPX/SIO_2_ as a substrate, with a single layer of graphene film inserted between the vertical structures as shown in Fig. 7. The high sensitivity arises from the excitation of the optical resonance between graphene and the one-dimensional photonic crystal. The sensor is highly sensitive to the Fermi energy level of graphene, the thickness and refractive index of the sensing medium, and has a maximum sensitivity of 407 RIU^−1^. Graphene-based biosensors or gas sensors have promising applications in the terahertz frequency range.

**Fig. 8:**
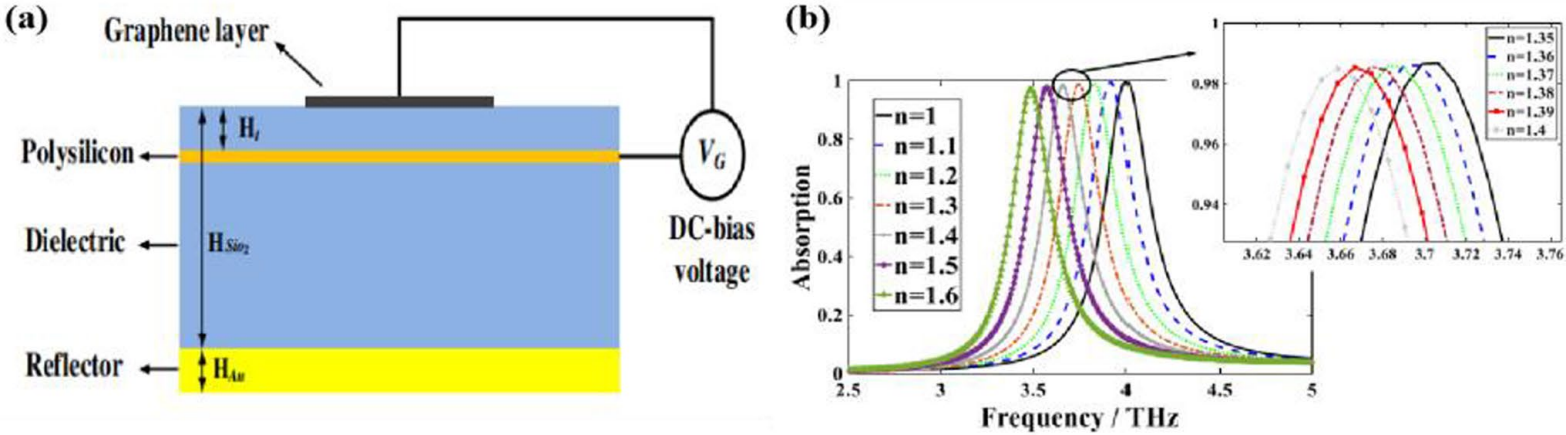
**a** Arrangement of the proposed structure and applying the external gate voltage VG. **b** Absorption spectra vs. the changes of the RI of the analyte. [84] Copyright 2022, Plasmonics

Nickpay et al. [84] proposed a folded split-ring structure-based metasurfaces graphene resonance sensor, which exhibits perfect absorption at 4 THz, with a *Q*-factor of 13.76, the resonance peak of refractive index sensitivity of the analyte to be measured, and sensitivity of 851 GHz/RIU results, as shown in Fig. 8. The current graphene metasurfaces biochemical sensors have been applied to a variety of biochemical molecules analysis, including medical, biomedical, environmental, security and defense fields.

### Graphene terahertz metasurfaces detects protein-like molecules

Graphene sensor devices with good selectivity, sensitivity and other advantages have attracted much attention [85, 86]. Graphene itself has a superior field enhancement effect, but the existence of curling, agglomeration, and interlayer stacking phenomenon is not conducive to the production and output of biosensing, through the combination of graphene and metasurfaces, the systemic action of graphene and metasurfaces, so biometric and biocompatibility can be further enhanced.

Proteins are the executing molecules that respond to instructions stored in the genetic material of any life form, and protein-targeted sensors can be made by immobilizing specific biomolecules on a surface [87]. However, it is difficult to control surface patterning or reversible deposition of target molecules, which would allow for reusable surfaces. Patel et al. [88] developed a tunable infrared metamaterial biosensor, which uses phase-change materials to detect hemoglobin and urine, and changes the phase through temperature changes to provide adjustable characteristics for biosensor applications, providing a reference method for biological detection. Proteins are selectively immobilized on graphene c-covered planar or porous surfaces with controlled orientation and surface density. The protein complexes can be predicted by assay to be important for identifying the structure and function of the cell.

**Fig. 9:**
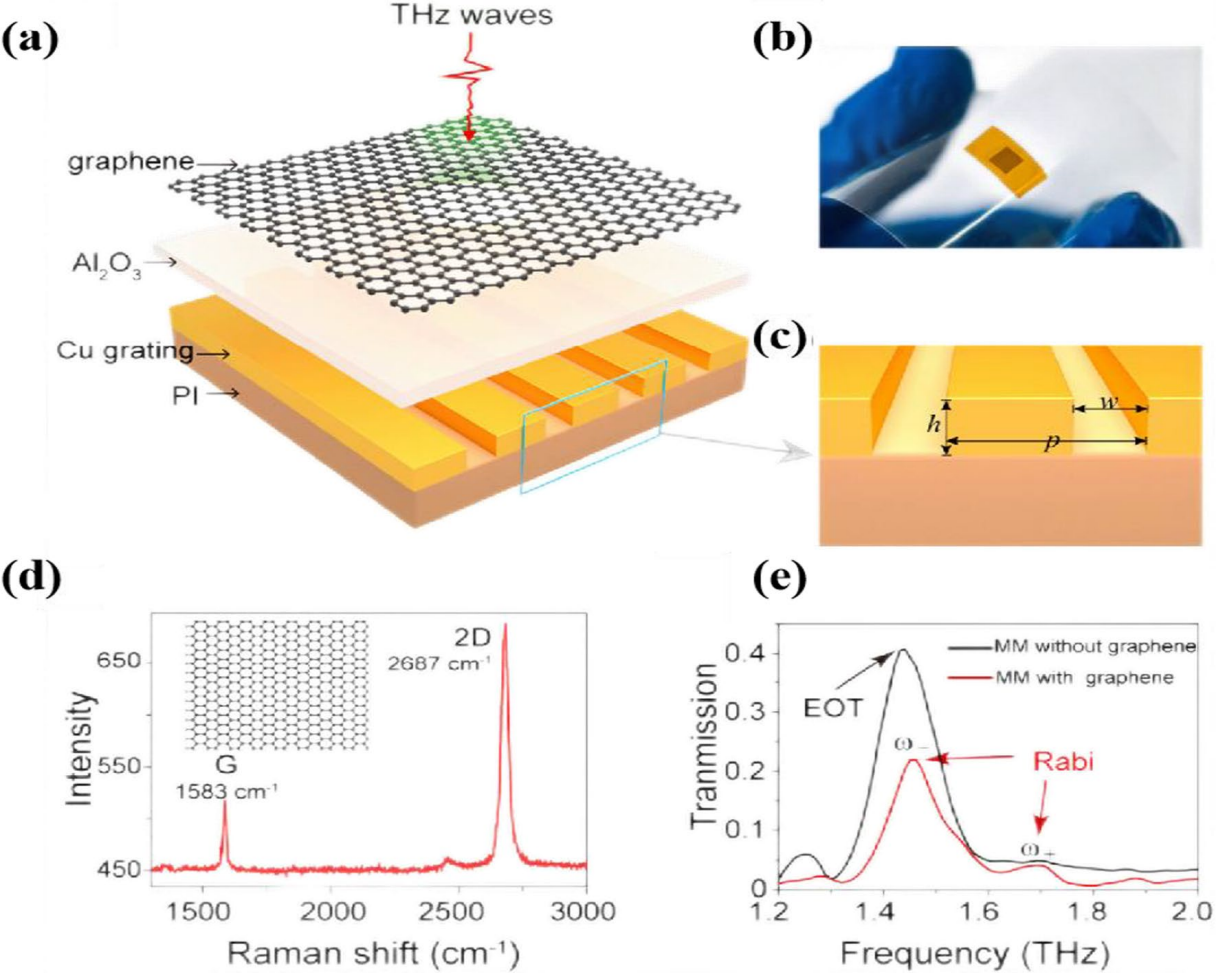
Terahertz metal–graphene hybrid metamaterial. **a** Schematic diagram of THz metal–graphene hybrid. **b** Image of the metamaterial sitting on PET substrate. **c** Zoom-in view of the cu grating structure. **d** Raman spectrum of the transferred monolayer graphene. **e** Experimental transmission spectra of the metamaterials with/with graphene. [89] Copyright 2022, Sensors and Actuators B: Chemical

**Fig. 10:**
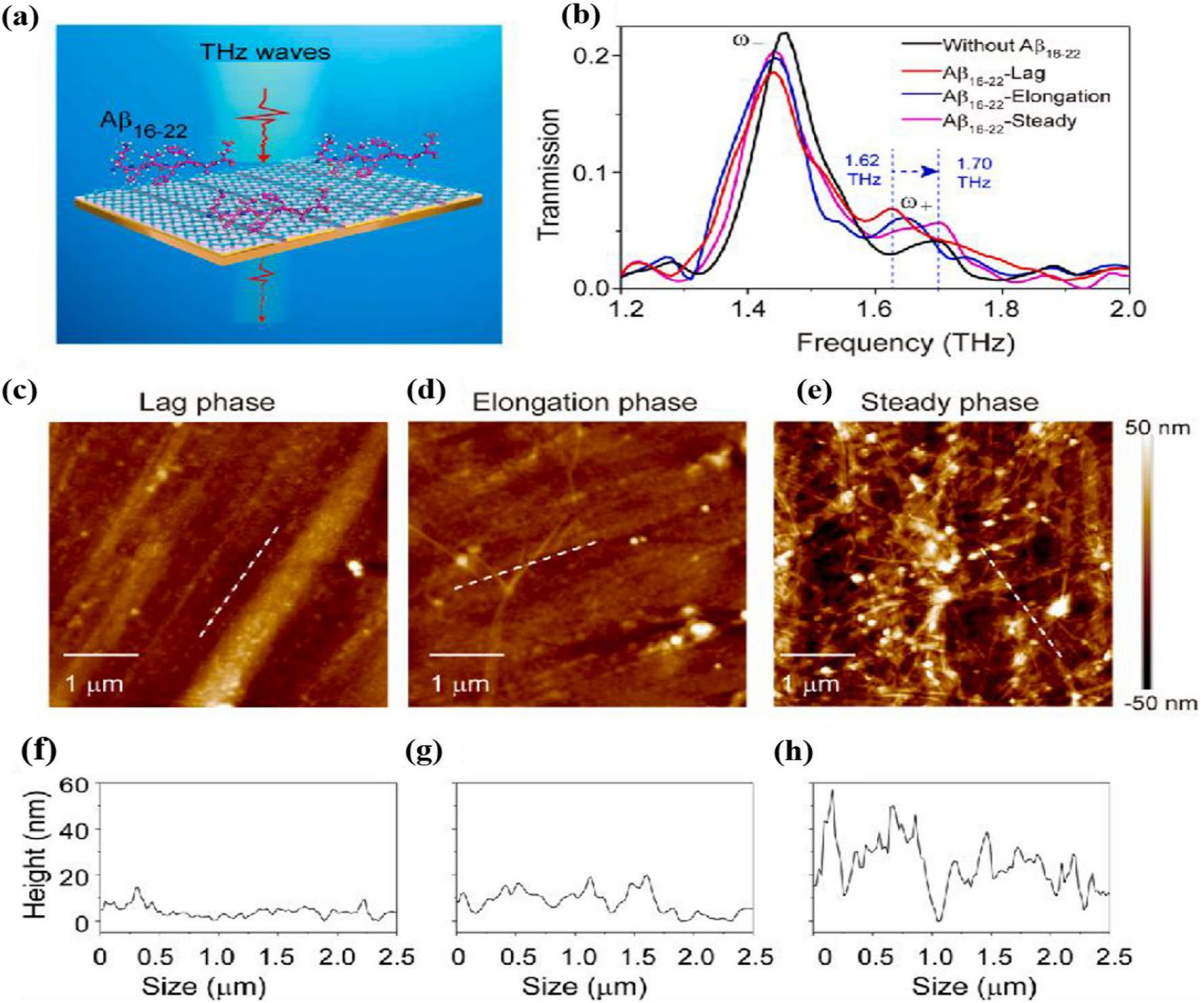
**a** Schematic illustration of metal–graphene hybrid metamaterial with Aβ_16–22_ peptides. **b** Experimentally measured THz transmission spectra of metamaterials without/with Aβ_16–22_ in different aggregation phases. **c**–**e** AFM images and **f**–**g** heights of Aβ_16–22_ aggregates in lag, elongation, and steady phases, respectively [89] Copyright 2022, Sensors and Actuators B: Chemical

The Aβ_16-22_ peptides are the main marker of Alzheimer's disease. Xu et al. [89] proposed a novel terahertz metal–graphene hybrid metamaterial to detect the fibrillation of Aβ_16–22_ peptides as shown in Fig. 9, which can well detect the aggregation process of Aβ_16–22_ peptides due to the change of the Fermi energy level leading to the change of the position of the Rabi splitting peak as in Fig. 10. As the electron doping of Aβ_16-22_ decreases, the Fermi energy level of *p*-doped graphene moves away from the Dirac point with the aggregation process, and the movement of the Fermi energy level of graphene is important due to the stacking of the *π*–*π* bond between Aβ_16-22_ peptides and graphene.

**Fig. 11:**
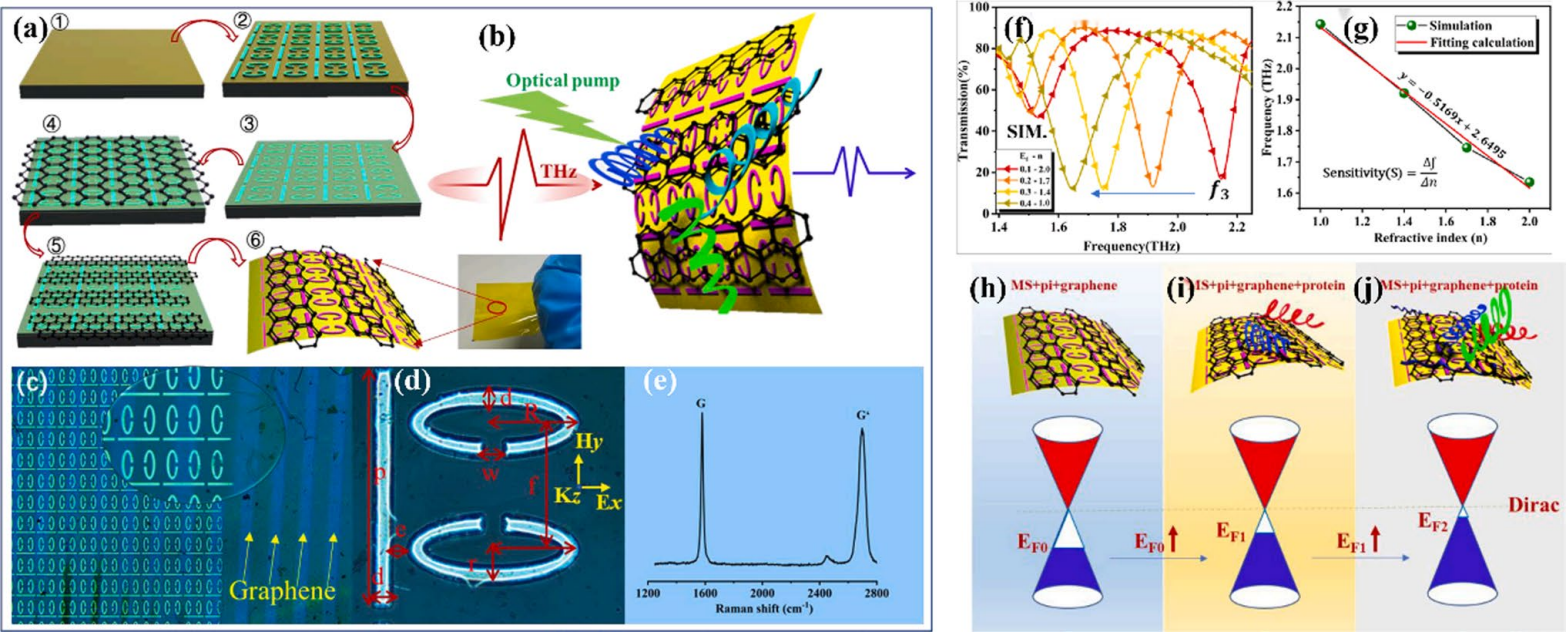
Fabrication and mechanistic analysis of PGP@EMS biosensor; Manufacture of the PGP@EMS biosensor. **a** Manufacturing process: ① A PI film was spin-coated on a quartz substrate; ② preparation of EIT-like metasurface on the PI film; ③ PI film was spin-coated on the metasurface; ④ graphene was transferred onto the PI film; ⑤ trilayer graphene was patterned into stripes; ⑥ by soaking the sample in hydrofluoric acid, a flexible-based THz biosensor was obtained. **b** Qualitative sensing of plant protein. **c** Optical microscope images of the samples. **d** The unit cell of the EIT-like metasurface. The corresponding parameters were: *p* = 70 µm, *c* = 8 µm, *w* = 6 µm, *r* = 10 µm, *f* = 40 µm, and *r* = 24 µm. **e** Raman spectrum of graphene. **f** y-polarized transmission curve with analyte refractive index increasing from 1.0 to 2.0 and Fermi level of graphene simultaneously increasing from 0.1 to 0.4. **g** *y*-polarized frequency shift at *f*_3_ for different refractive indices of analyte and different Fermi levels of graphene, extracted from (**h**–**j**) Change of Fermi level with patterned graphene mechanisms in the presence of plant protein. [19] Copyright 2022, Results in Physics

Yao et al. [19] developed a novel flexible terahertz biosensor as shown in Fig. 11, which is composed of electromagnetically induced transparent-like metasurfaces and patterned graphene for multidimensional ultrasensitive detection of plant proteins, with a detection limit of 42.3 pg/mL. For plant protein molecules based on the change of frequency phase, by the result of the shift of the Fermi energy level of graphene to the Dirac Point and the change of the Fermi energy level theoretical analysis to interpret the sensing mechanism.

**Fig. 12:**
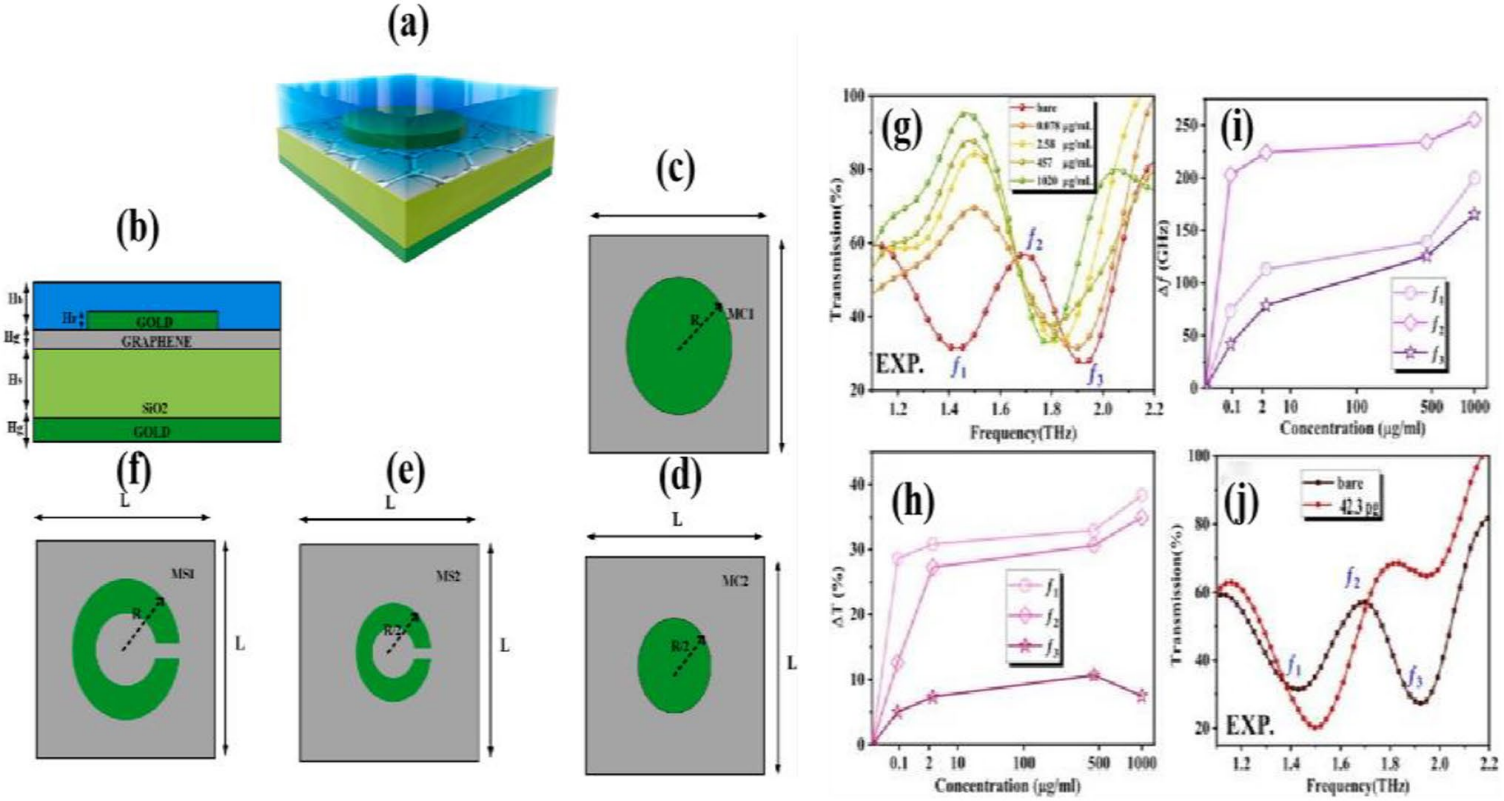
**a**–**f** Graphene-based biosensor with circular and split-ring resonator metasurface, and **g**–**j** absorption response of graphene-based biosensor circular metasurface and split-ring resonator metasurface. [90] Copyright 2022, Physica B: Condensed Matter

Parmar et al. [90] proposed a graphene metasurface refractive index biosensor, as illustrated in Fig. 12 for the detection of hemoglobin and urine biomolecules, analyzing the metasurface in the form of circular and split-ring resonant cavities. It also analyzes the different metasurface sizes, varies the thickness of different physical layers, and captures biomolecules by exploiting the compatibility of the graphite surfaces combined with biomolecules. A highly sensitive sensor has been developed for the detection of medicinal hemoglobin and urine biomolecules.

Metasurfaces learned by this method allow the detection of other components such as metal ions, carbon dioxide, other aromatic molecules, etc. [84]. Therefore, the carrier mobility of graphene can be changed by changing the surface. The kinetic and Raman spectroscopy results are in good agreement with the numerical simulation results. It developed a novel detection tool for disease diagnosis.

### Graphene terahertz ultra-surface detection of nucleic acids

DNA is a macromolecular polymer composed of deoxyribonucleic acid. It is a biomolecule of biological genetic information. It is very critical for the detection of biological macromolecules to provide information on living individuals, and DNA biosensors are widely used in different fields of clinical medical diagnosis, environmental detection, and drug development [91, 92]. There is a fingerprint between DNA and terahertz waves, through the combination of graphene and ultra-surface, which cannot change DNA detection without changing the stability and biological activity of DNA.

**Fig. 13:**
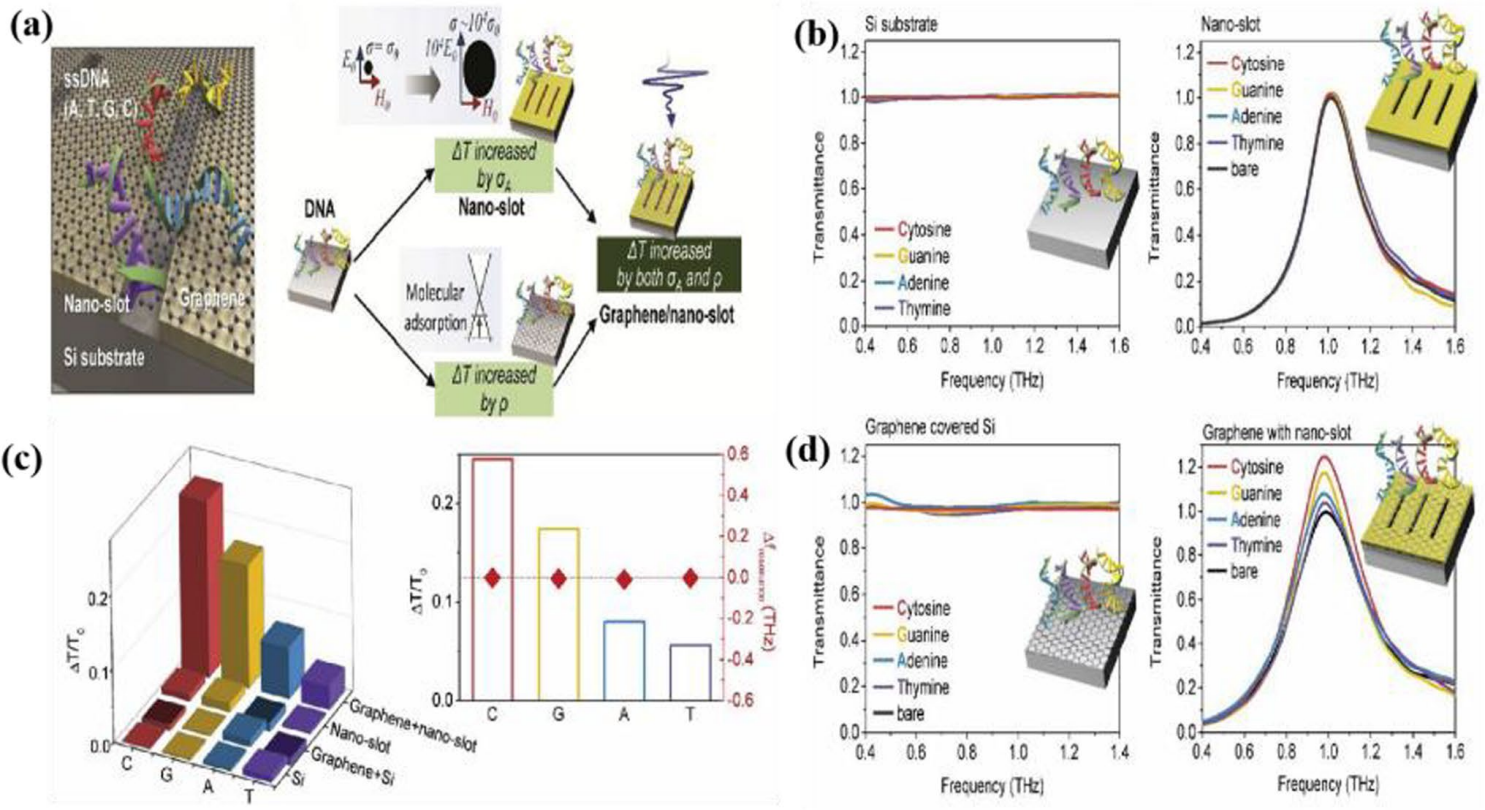
DNA adsorption on the graphene-combined nano-slot metamaterial. **a** The THz sensing concept is used in this study and the black circle denotes absorption cross section, *σ*, with and without absorption enhancement which is proportional to *Ex*/*Hy*. **b** Change in THz transmission spectra due to ssDNA on Si, a bare nano-slot metamaterial, graphene-covered Si. **c** Summary of the change in transmittance for the four sensing platforms, and graphene-covered nano-slot metamaterial, respectively. **d** The spectra are normalized to the transmittance for bare Si and maximum transmittance at the resonance frequency of the nano-slot metamaterial (1.0 THz). [19] Copyright 2020, Sensors and Actuators B: Chemical

By designing graphene-combined nano-grooves with terahertz metasurfaces as shown in Fig. 13, Lee et al. [93] designed graphene-combined nano-slots combined with terahertz metasurfaces; the resonance frequency and terahertz electric field strength are enhanced by combining the resonance structure of graphene and tuned photoelectric properties; the adsorption cross section of graphene toggles with target molecules ssDNA is significantly increased by *π*–*π* stacking with DNA molecules tightly bound; the nano-slot resonance assisted by strongly enhanced and aggregated terahertz waves, even in the presence of nmol/mm^2^ levels, can be observed [94]. More DNA molecules can be observed, resulting in highly sensitive DNA biosensors.

**Fig. 14:**
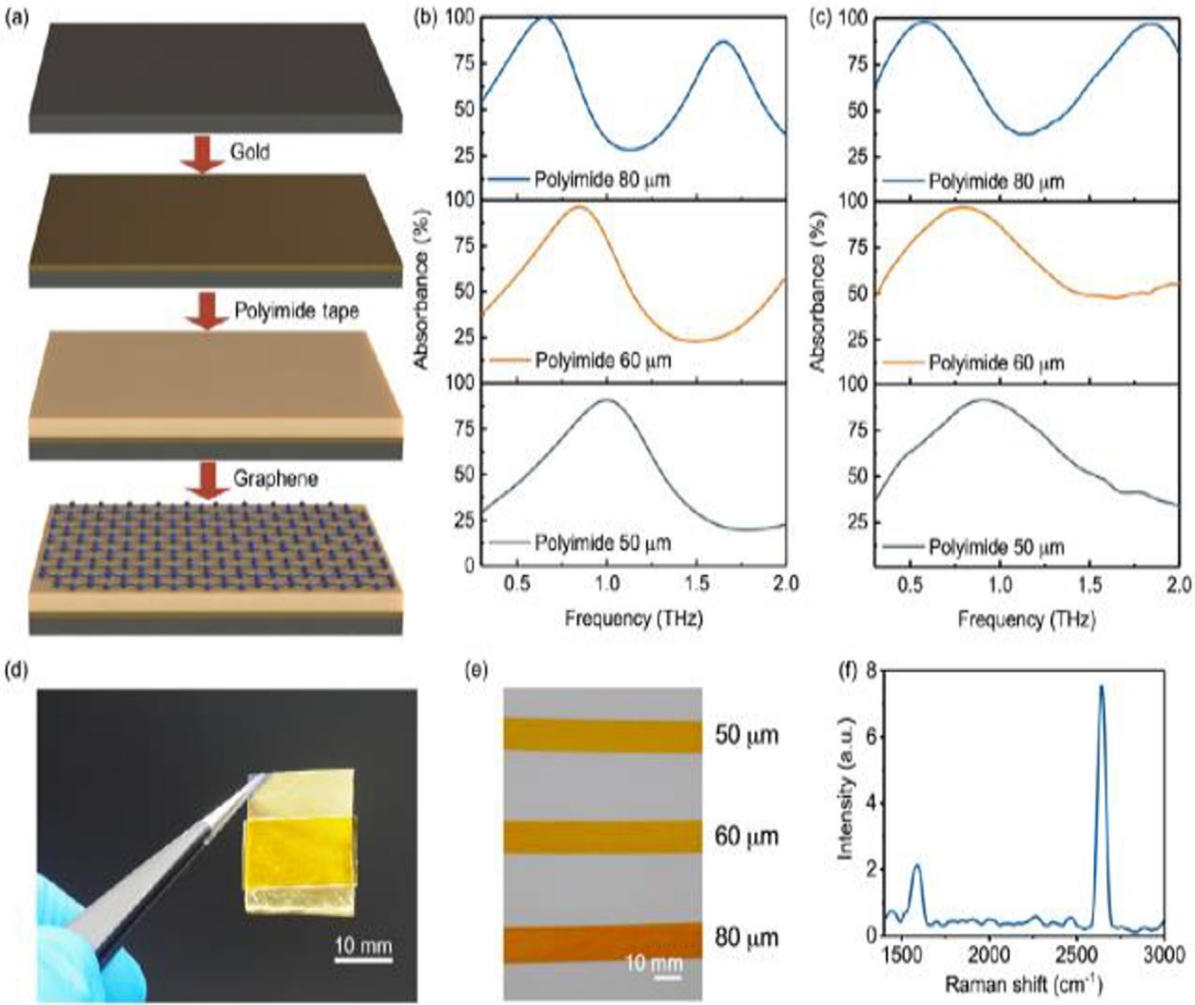
Sensing principle of graphene sensor operating in the THz regime. [95] Copyright 2020, ACS Applied Materials & Interfaces

**Fig. 15:**
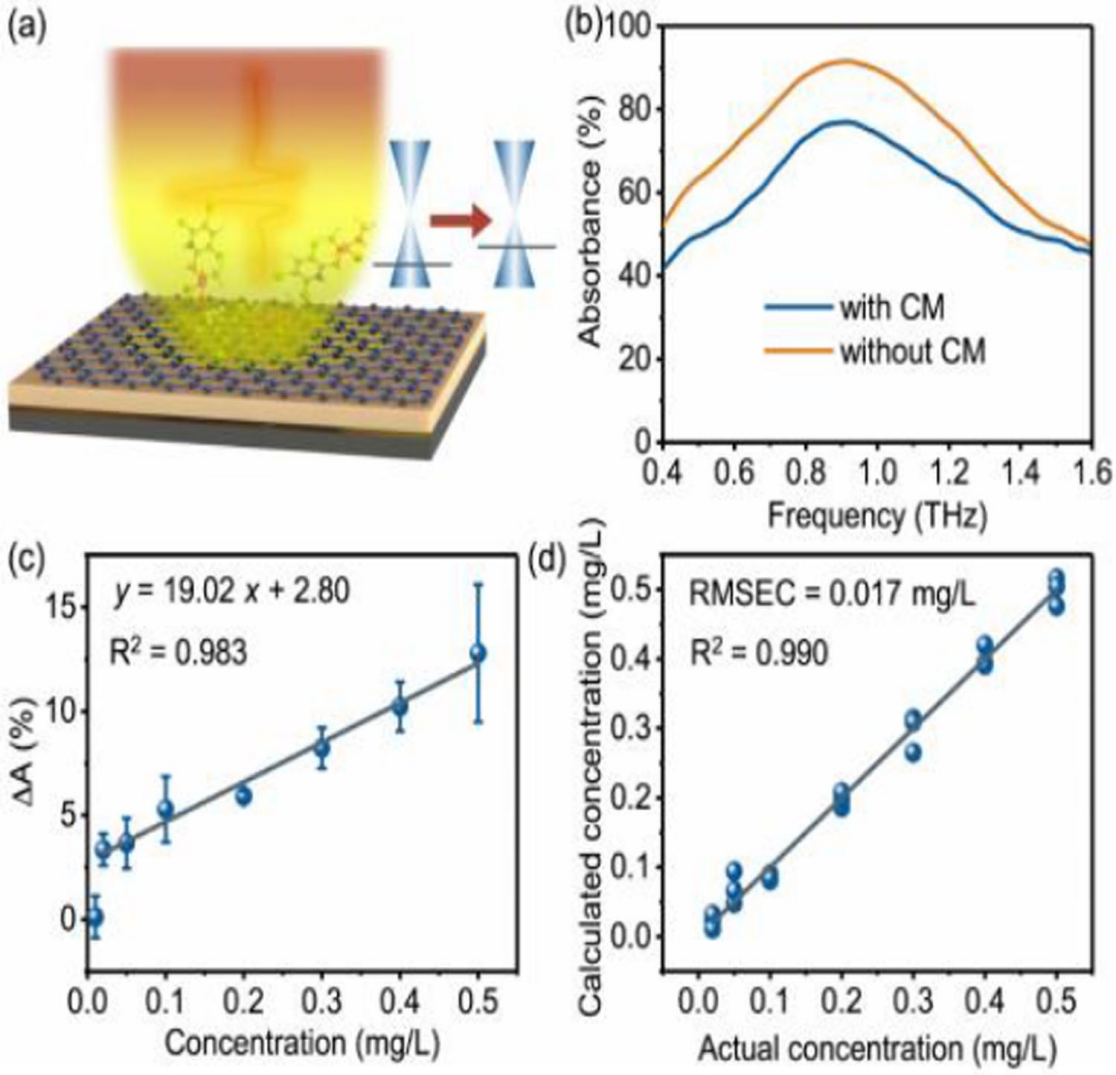
Sensing chlorpyrifos-methyl using graphene sensor. **a**, Schematic of a graphene sensor for reflective sensing of chlorpyrifos-methyl. **b**, Experimental absorption curves for the graphene sensor with/without chlorpyrifos-methyl (CM is short for chlorpyrifos-methyl). The concentration of chlorpyrifos-methyl was 0.50 mg/L. **c**, Absorbance changes when chlorpyrifos-methyl molecules are adsorbed at concentrations from 0.01 to 0.50 mg/L. **d**, PLS regression result of chlorpyrifos-methyl molecules at concentrations from 0.02 to 0.50 mg/L. [95] Copyright 2020, ACS Applied Materials & Interfaces

Xu et al. [95] the application design of graphene on the metasurface uses silicon polymer gold as the substrate and graphene as the binding layer on the metasurface, as shown in Fig. 14. Simulation by FDTD, the performance of the sensor depends on the Fermi energy level, the refractive index of the analyte, the thickness of the analyte and the variation of absorption coefficient to change the terahertz absorption, and successfully detected chlorpyrifos using this sensor. As shown in Fig. 15, the detection line is 0.13 mg/L by detecting in real sample concentrations up to 0.60 mg/L of pesticide molecules. This flexible sensor demonstrated high stability in 1000 bending cycles. These results indicate the easy fabrication, flexibility, and multipurpose characteristics of graphene thin film metasurfaces, utilizing the promotion in detection.

**Fig. 16:**
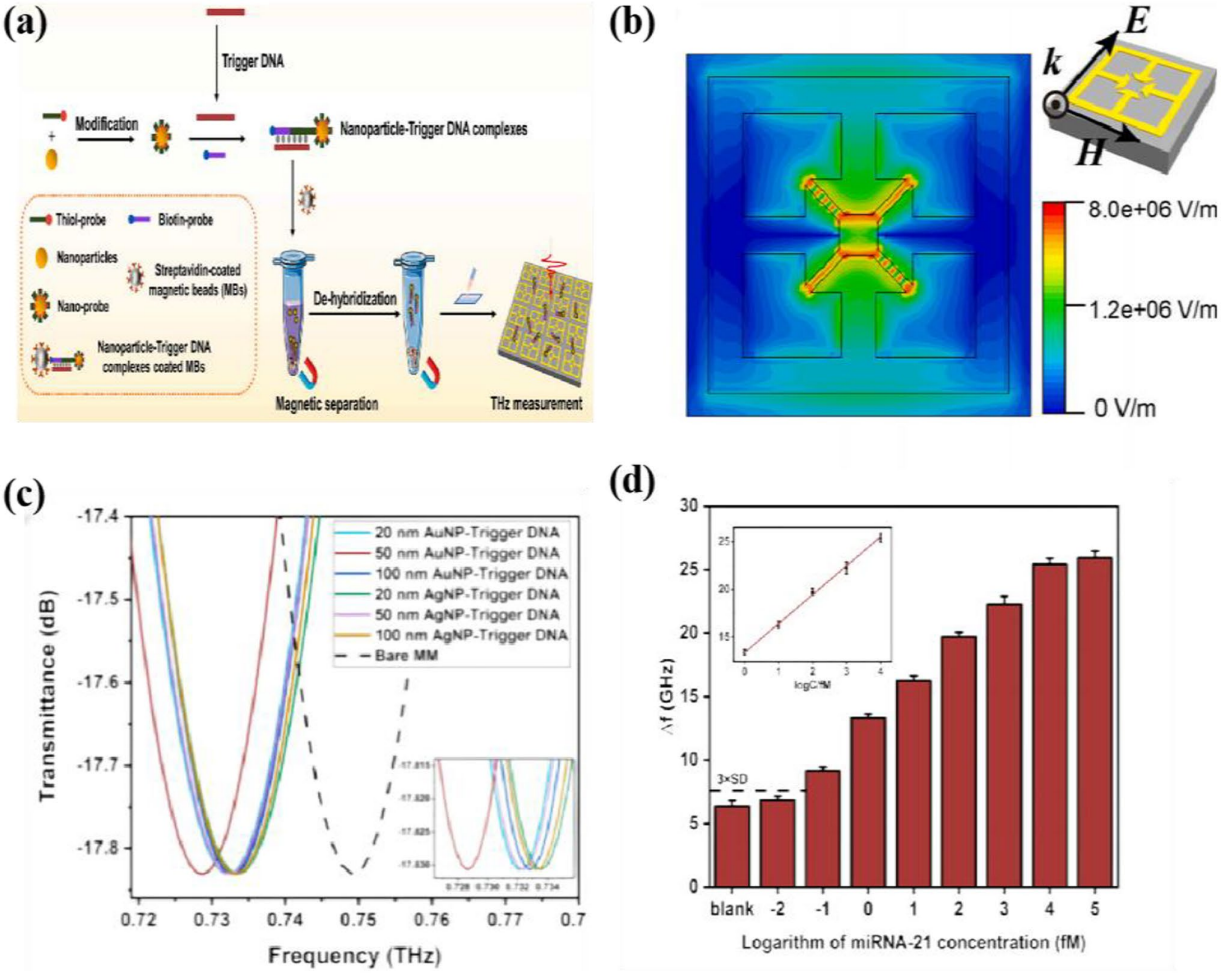
Schematic **a** description of THz metamaterial biosensor for miRNA detection, **b** Sensitivity of THz metamaterial biosensor for miRNA-21 detection. The inset graph shows the linear relationship between Δ*f* and the logarithm of miRNA-21 concentration. Error bars indicate the SD (*n* = 3). **c** Electric field distribution at the resonant frequency. Error bars indicate the SD (*n* = 3). **d** Sensitivity of THz metamaterial biosensor for miRNA-21 detection. The inset graph shows the linear relationship between Δ*f* and the logarithm of miRNA-21 concentration. Error bars indicate the SD (*n* = 3). [96] Copyright 2021, Biosens Bioelectron

Yang et al. [96] the proposed a method for sensitive detection of miRNA based on the displacement amplification technique, in which chain displacement amplifies the target miRNA and generates secondary DNA molecules, and by combining with nanoparticles the binding of these complexes generates a metasurface frequency shift that exhibits good detection sensitivity under optimal conditions. Novel terahertz biosensors with potential applications in nucleic acid analysis and cancer diagnosis are demonstrated, as shown in Fig. 16.

**Fig. 17:**
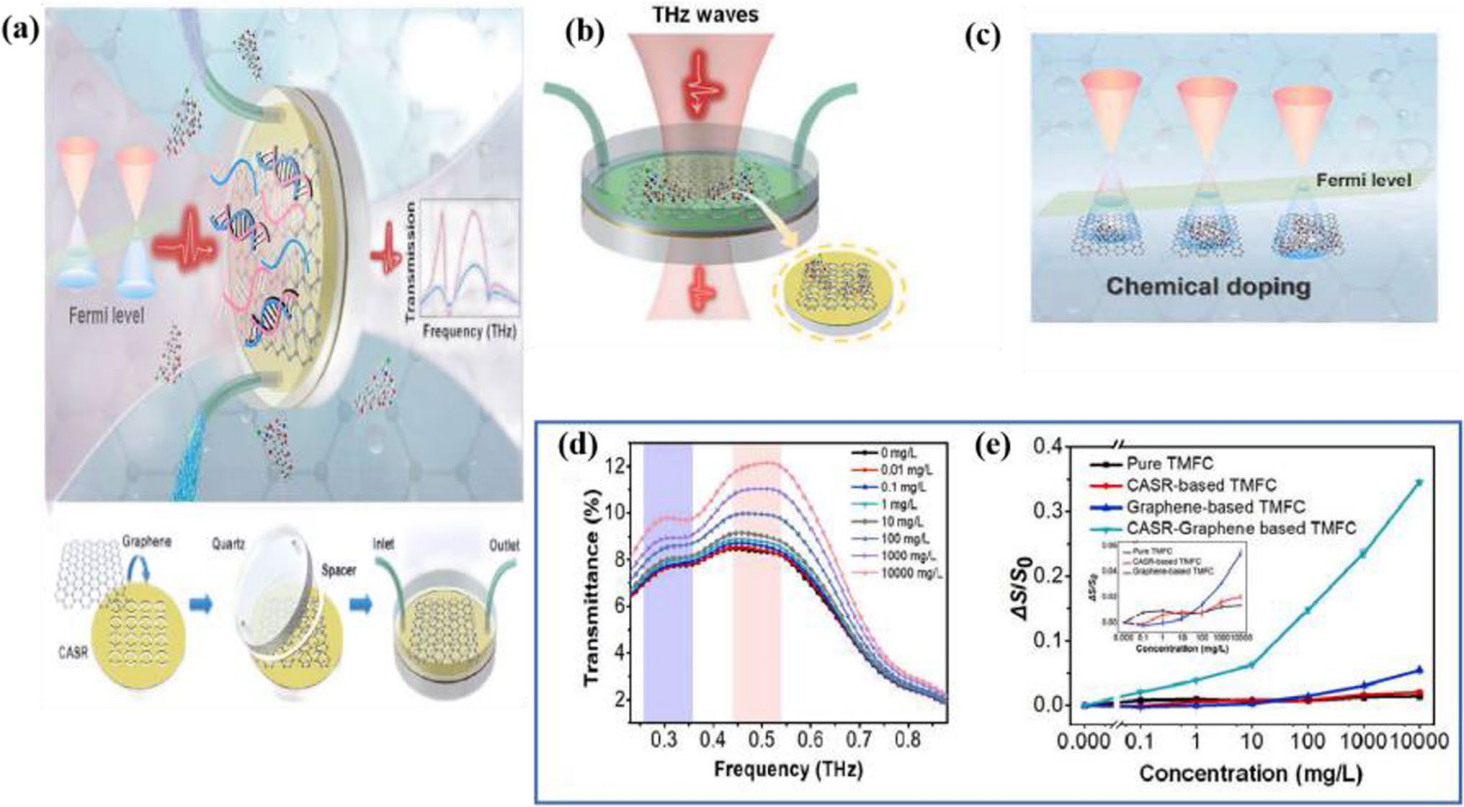
The CASR-graphene-based THz microfluidics platform. **a** Schematic drawing showing the working principle and design of DNA biosensing based on the proposed CASR-graphene THz microfluidic cell; **b** Schematic diagram of the manufacturing flow for the CASR-graphene THz microfluidic cell. **c** Schematic illustration of the position of the Dirac point and the Fermi level as a function of chemical doping. **d** Measured THz transmission spectra of DCH aqueous solutions at different concentrations. **e** Relative change rate in effective transmission areas of the four types of TMFCs for DCH aqueous solution detection versus DCH concentrations. The inset is an enlarged view of the relative change rate in effective transmission areas. [69] Copyright 2021, Biosens Bioelectron

Meanwhile, metasurfaces-assisted terahertz real-time label-free biosensors have attracted extensive research, and high sensitivity in the terahertz range is still challenging for specific detection of highly absorbing liquid samples or water molecular liquids, water has strong absorption of terahertz waves, to overcome these drawbacks researchers have used a microfluidic technique combined. Zhou et al. [69] developed terahertz metasurfaces of graphene doped into the microfluidic cavity for sensitivity detection as shown in Fig. 17. The combination of the two can effectively reduce the sample volume of the sample and improve the interaction of terahertz waves with biomolecules. Thus, the sensitivity of detection is provided, and biomolecule detection is performed on this basis through a comparative study of the structure, and specific aptamers are modified on the graphene surface, thus enabling the identification and detection of *E. coli* O157:H7 first. Selective detection of 100 nM DNS solution was achieved. Through mechanistic analysis due to n-type molecules doped by non-covalent surface, including van der Waals forces, *π*–*π* stacking, and hydrogen bonding. The *p*-type doped graphene Fermi energy levels move to the Dirac point, in both structures graphene on the metasurfaces of a large number of molecules will move the Fermi energy level to the Dirac point, resulting in the formation of the original graphene has a lower photoconductivity, thus higher transmittance and microfluidics binding to provide highly sensitive detection [97].

As an efficient platform for biosensing in liquid environments, it can selectively and label-free detect biomolecules. By changing the corresponding DNA sequence, the sensor can be altered to detect other different DNA molecules. It results in that the graphene-bound terahertz metasurface platform not only affords a broad research field for future medical applications but also proposed theoretical support for the optoelectronic effects of thin films such as 2D materials.

### Graphene biosensor detects chemical molecules

**Fig. 18:**
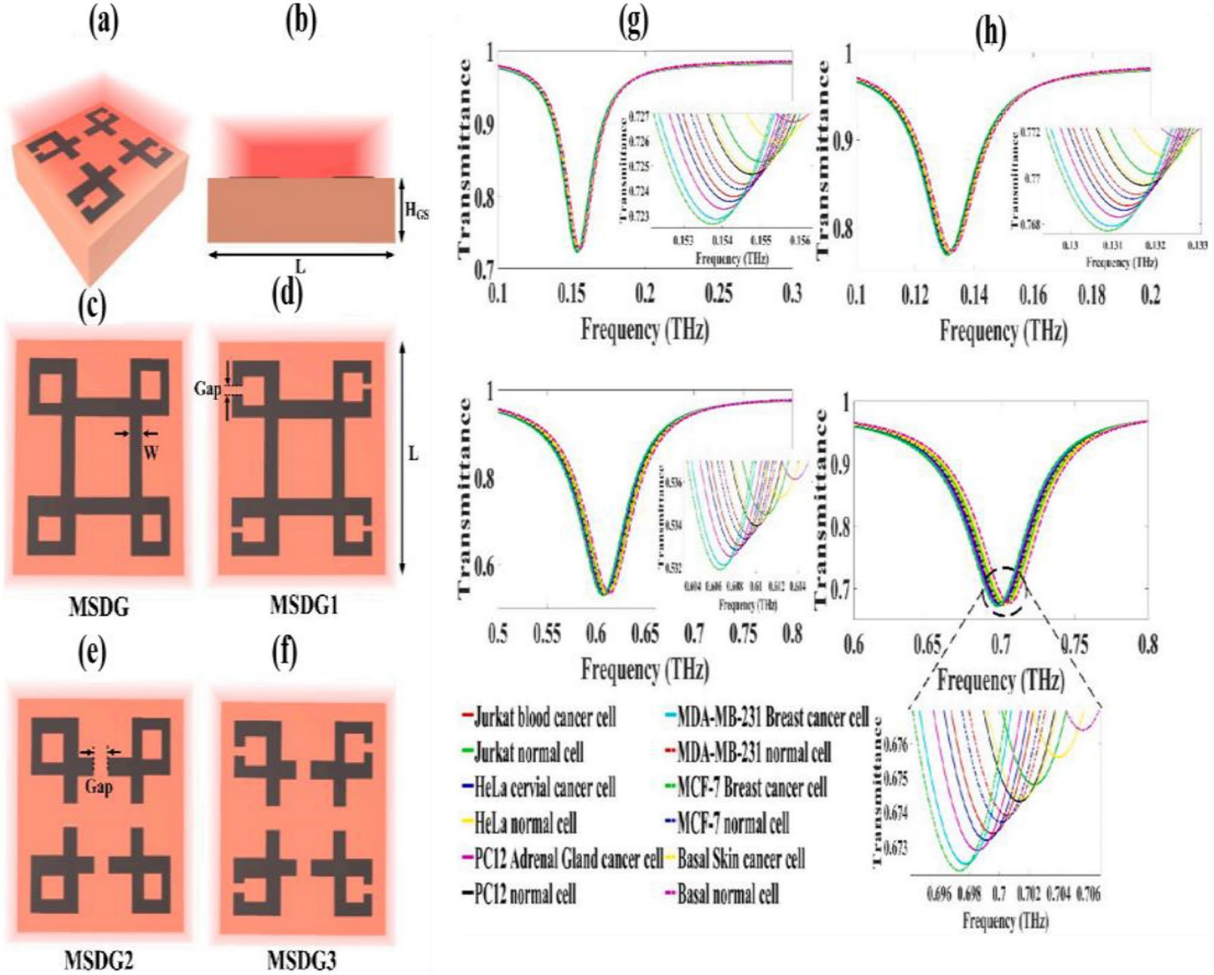
Detection of various cancer with their normal cell for proposed sensor. **a** 3D view, **b** front view, **c** overhead view of metasurface senor design with no gap, **d** overhead view of metasurface senor design with gaps at the outer boundary, **e** overhead view of metasurface senor design with gaps at inner boundary, **f** overhead view of metasurface senor design with gaps at both outer and inner boundary. **g** and **h** Cancer cell detection performance and linearity. [98] Copyright 2022, Diamond and Related Materials

Terahertz propagating in free space interacts weakly with the sample and is not sufficient for trace detection. By transferring graphene to the sample surface, the metasurfaces absorber constructs a graphene metasurfaces absorber heterogeneous structure as shown in Fig. 18, and the terahertz time-domain spectroscopy system is used to simulate the transmission spectrum of the resonant cavity to detect antibiotics by observing the frequency shift of the frequency, and its sensitivity is improved by one order of magnitude. The lower detection limit concentration was 0.02 mg/L [98].

**Fig. 19:**
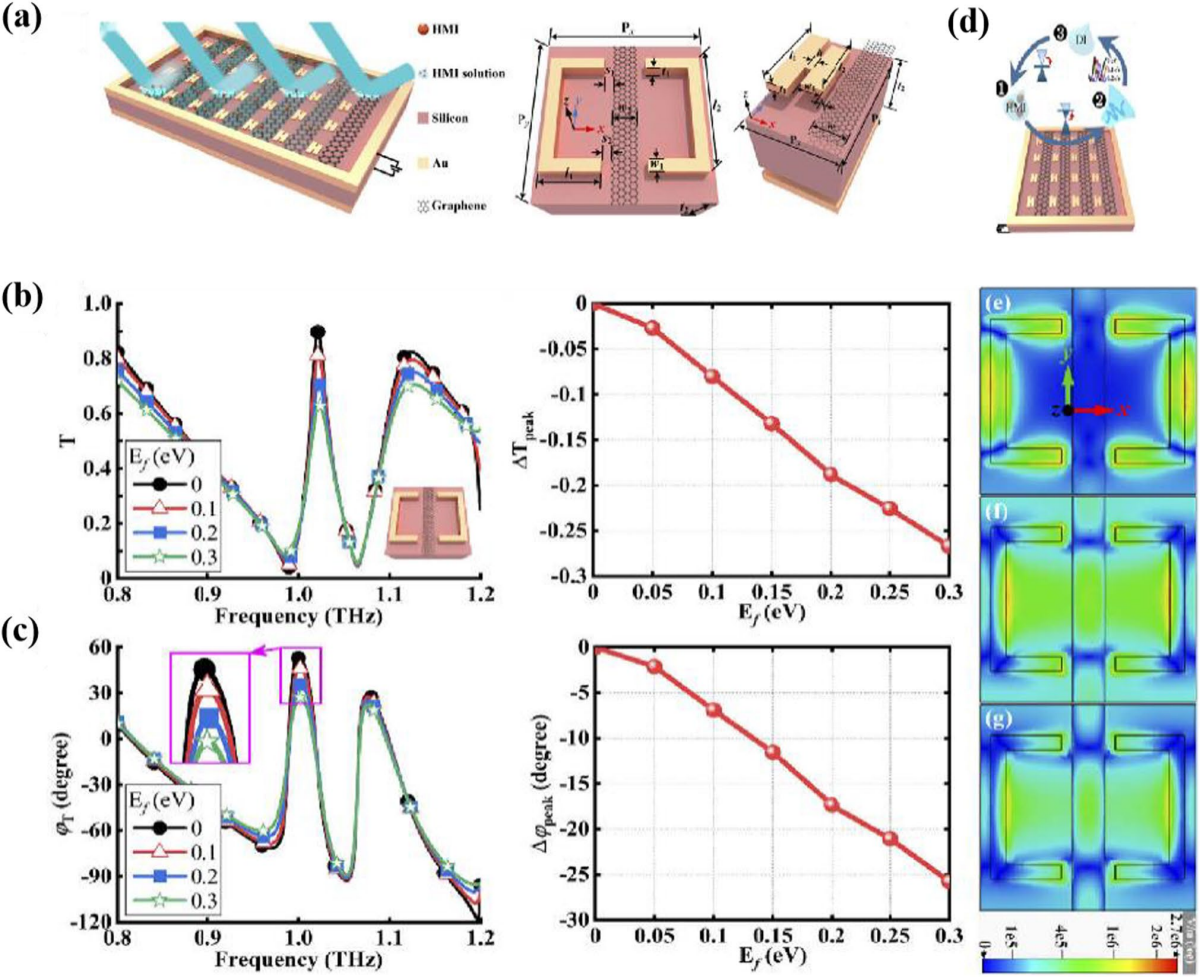
**a** Designed reflective graphene metasurface to detect metal ions HMI detection based on GTM, transmission-type, and refection-type structures. **b** Amplitude difference **c** phase difference peak graphene transmission coefficient graphene transmission fermi energy resonance peak changes, **e** electric field distribution at the transmission resonance peak. [99] Copyright 2021, Optical Materials Express

The accurate and rapid detection in the terahertz band of this study contribute a novel mean of detection. Zhao et al. [99] proposed a graphene-based terahertz metasurfaces approach to detect heavy metal particles as shown in Fig. 19 to design reflective metal ion detection metasurfaces devices. The proposed concept and the approach of detection help to design the desired highly sensitive, fast and reusable graphene terahertz biochemical sensors through selective adsorption and functional group-specific selectivity on graphene terahertz metasurfaces.

**Fig. 20:**
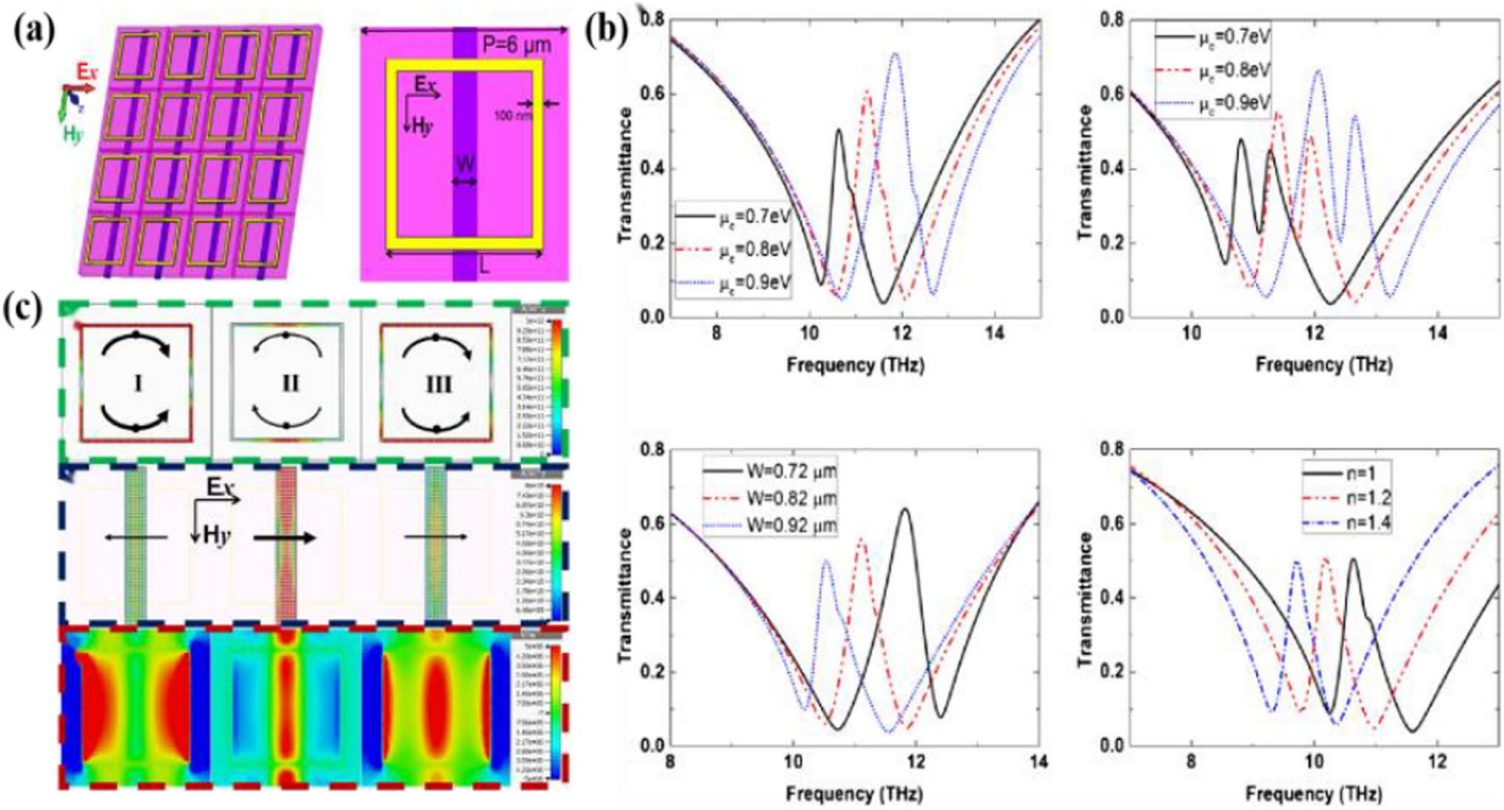
Schematic of said hybrid structure, **a** schematic diagram of the cells of said structure, **c** Transmission spectra of various hybridized structures, **b** Graphene surface current distribution and magnetic field distribution. [100] Copyright 2015, Plasmonics

Ding et al. [100] developed a novel metal and graphene hybrid metasurface hybrid sensor consisting of a graphene grating and a square closed-loop resonator split by a dielectric substrate as shown in Fig. 20. The spectral coordination caused by the change of the Fermi energy level of graphene and the performance effect of refractive index sensing were analyzed. The experimental results were analyzed for the change of refractive index 1 of the analyte to 1.4 and the offset of the resonance peak to 2 THz.

**Fig. 21:**
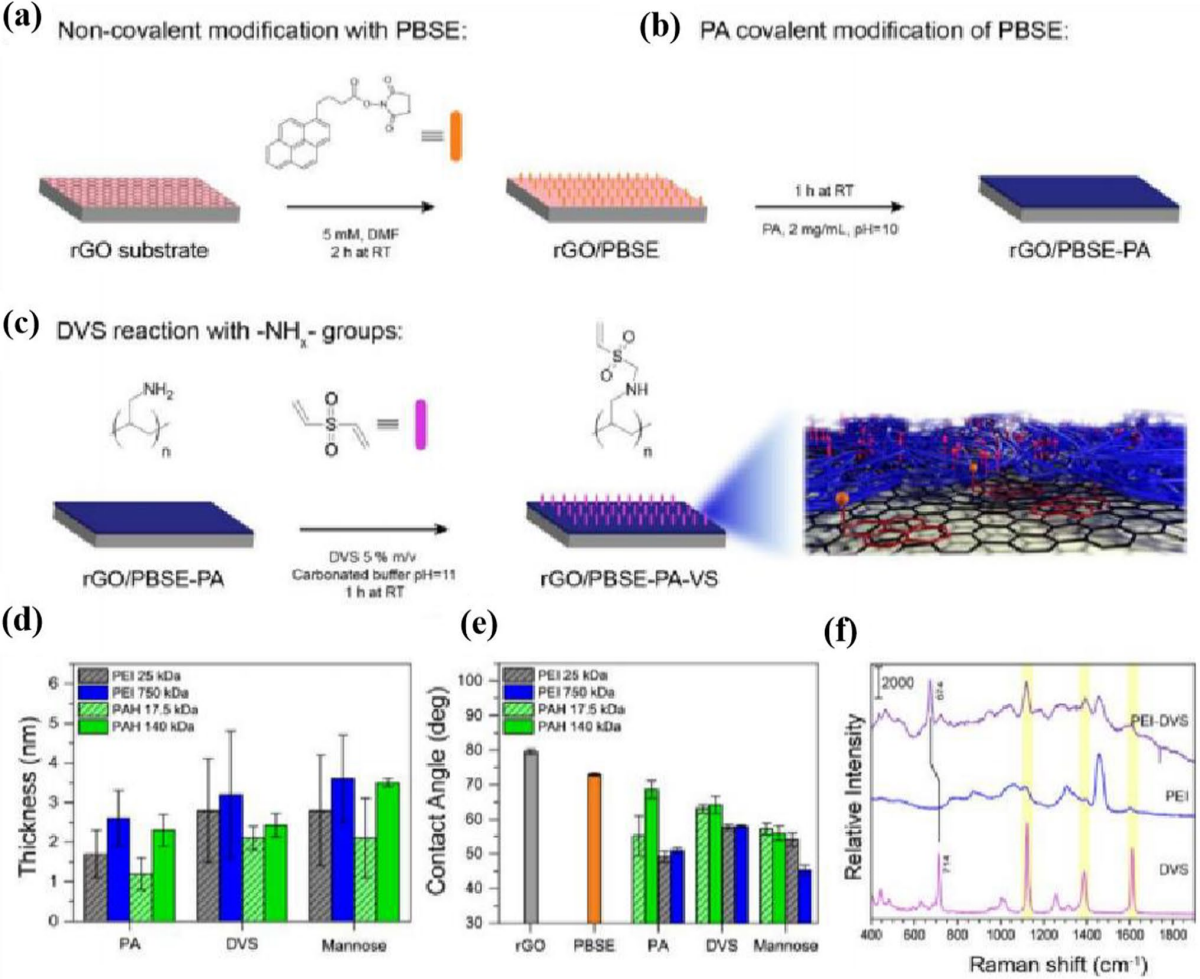
**a**–**c** Schematic representation of the functionalization steps for obtaining PA-VS surfaces. **d** Thickness and **e** contact angle evolution after different functionalization steps: PA anchoring on rGO/PBSE surfaces; changes after DVS functionalization (DVS); mannosylation (Mannose). Error bars represent the 95% confidence interval. **f** Raman spectra of DVS, PEI (750 kDa), and the product of the reaction between them in aqueous solution (PEI-DVS). [101] Copyright 2021 ACS Applied Materials & Interfaces

A bifunctional covalent scaffold for modification with metasurface functionalization was reported by Esteban Piccinini et al. [101] as shown in Fig. 21. The structure consists of surface engineering of graphene by a heterobifunctional supramolecular covalent scaffold based on vinyl sulfonated polyamines (PA-VS). The structure has a high sensitivity due to the effective confinement of the plasma. This is combined with the low conductivity of graphene the structure has a strong chemically functionalized modification with a high affinity for lectins. The resistance to non-specific targets was increased by nearly 7 times. The proposed structure can be used for chip detection sensing applications and is capable of multiplexing optical detection species for multi-split analysis and detection [102]. The problem faced by the terahertz biochemical molecule detection involved so far is that the stability of the biochemical molecules of interest remains a challenge for applications. Repeated use in complex sample matrices as well as under environmental conditions should be key factors affecting the stability of graphene metasurface sensing. The need to develop functional devices for stability detection is a top priority in current biochemical assays.

### Graphene nanomaterials into metamaterial devices

The graphene nanomaterials transformed metasurfaces devices have gained the attention of researchers. Graphene metasurfaces heterostructures become a research thermoelectric for terahertz wave modulators [103, 104]. Graphene doping induced by external voltage helps to improve the modulation capability, and more and more two-dimensional materials can be introduced into the terahertz metasurfaces sensing technology, while the introduction of nanomaterials can increase the sensitivity of detection, such as; gold nanoparticles, carbon Materials, quantum dots etc., are widely used in the field of sensing. For example, gold can be functionalized to incorporate biomolecules, which will bring fruitful realization by introducing it to the field of terahertz sensing and may provide innovative research avenues for new terahertz detection. Table 2 lists examples of the use of terahertz ultra-surface combined with materials in recent years to achieve the development of interdisciplinary studies, performance, and characteristics of terahertz ultra-surface sensing technology, including the core materials for the combination of ware [85, 105–116]. We can see that terahertz sensing presents miniaturization, trace, specificity development, and the combination of new materials, new technologies, and new methods combined to achieve a low-cost, efficient, specificity practical means of detection. It is expected to be promoted to commercial applications and shows application prospects.

**Table 2:** Comparison of various terahertz ultra-surfaces for biochemical sensing

Ultra-surface core materials	Function	Performance	References
Carbon nanotubes	2,4-Dichlorophenoxyacetic acid	2.0 × 10^–3^/ppM	[105]
Metal	Citrate	1 mM	[106]
Metal	Glioma cells	248.75 kHz/cell /mL	[85]
Metal	Carcinoembryonic antigen	20 ng/mL	[107]
All-metal construction	Bovine serum protein	0.035 mg/mL	[108]
Silicon	Cancer cells	104 cells/mL	[109]
Silicon	DNA detection	0.05 ng/uL	[110]
Silicon	Antibody/Antigen detection	5 pg/mL	[111]
Material—two-dimensional	Chlorpyrifos methyl	2.2 ng	[112]
Gold	SARS-CoV-2 virus protein	4.2 fm	[113]
All-dielectric metasurface	Sensing microcystin-LR	0.002 ug/L	[114]
Metal grid	Doxycycline hydrochloride	1 mg/L	[115]
Graphene	DNA detection	100 nM	[116]

Although there are many reports on graphene terahertz ultra-surfaces, there are still many difficulties to develop from the research field to the commercial field, including the detection limit of graphene terahertz ultra-surfaces in practical analysis, the stability of biochemical molecules, and the hysteresis of biomolecule detection [19]. Its non-specificity, signal amplification, and other processes still need further research and exploration. There is still a requirement to explore the application of two-dimensional materials in ultra-surfaces, explore the development of more sensitive and stable terahertz functional devices, build new detection methods, etc.

## Conclusions

In summary, the combination of metasurfaces and optical matter obviously enhances the effect of becoming the focus of research on terahertz sensing technology. The development of devices using graphene metasurfaces has made substantial progress. The research using graphene metasurfaces as a starting point using different substrates and materials combinations has significantly enriched the relevant research results. The application of graphene terahertz sensor devices faces many challenges, and we need precise control of graphene generation, homogeneity, and surface functionalization. Current research on terahertz sensing detection technology requires two enhancements. The first of which is the design of the super-surface unit structure, and the reasonable selection of the substrate material is crucial for enhancing terahertz sensing to obtain stronger resonance properties. The second is the effective binding of the molecule to be measured to the target molecule by combining functionalized structural materials and optimization of analytical conditions. This is done while reducing terahertz loss to enhance the sensitivity of detection. However, it is promising that many graphene terahertz devices have achieved fast response and selectivity. Sensing technology is the interconnection of multidisciplinary technologies. With the development of novel spectroscopic techniques, data analysis algorithms, and materials science fields, technological advances are expected to promote the flourishing of biochemical sensing in biochemical detection, device performance, and applications in the foreseeable future.

## Data Availability

All data generated or analyzed during this study are included in this published article.

## References

[1] Wolfson AR, Mancini CM, Banerji A, Fu X, Bryant AS, Phadke NA, Shenoy ES, Arman W, Zhang Y, Blumenthal KG (2021). Penicillin allergy assessment in pregnancy: safety and impact on antibiotic use. J Allergy Clin Immunol Pract.

[2] Meng D, Liu J, Chen W, Cheng Y-Y, You K-W, Fan Z-C, Ye Q, Huang P-H, Chen Y-S (2022). Study on the enhancement mechanism of terahertz molecular fingerprint sensing. Results Phys.

[3] Cao Y, Cheng Z, Wang R, Liu X, Zhang T, Fan F, Huang Y (2022). Multifunctional graphene/carbon fiber aerogels toward compatible electromagnetic wave absorption and shielding in gigahertz and terahertz bands with optimized radar cross section. Carbon.

[4] Hao X, Li J, Zheng C, Li J, Yue Z, Tang X, Tan Q, Zhang Y, Yao J (2022). Optically tunable extrinsic chirality of single-layer metal metasurface for terahertz wave. Opt Commun.

[5] Chen T, Tang Z, Hu C (2022). The combination of terahertz spectroscopy and density functional theory for vibrational modes and weak interactions analysis of vanillin derivatives. J Mol Struct.

[6] Saito S, Inerbaev TM, Mizuseki H, Igarashi N, Note R, Kawazoe Y (2006). Terahertz phonon modes of an intermolecular network of hydrogen bonds in an anhydrous β-d-glucopyranose crystal. Chem Phys Lett.

[7] Sun M, Maqbool E, Han Z (2022). Terahertz sensing with high sensitivity and substance identification capability using a novel High-quality resonance supported by a thin structured silicon film. Opt Laser Technol.

[8] Picano B, Fantacci R (2022). Human-in-the-loop virtual reality offloading scheme in wireless 6G Terahertz networks. Comput Netw.

[9] Jiang Y, Li G, Ge H, Wang F, Li L, Chen X, Lv M, Zhang Y (2022). Adaptive compressed sensing algorithm for terahertz spectral image reconstruction based on residual learning. Spectrochim Acta Part A Mol Biomol Spectrosc.

[10] Wu X, Tao R, Zhang T, Liu X, Wang J, Zhang Z, Zhao X, Yang P (2023). Biomedical applications of terahertz spectra in clinical and molecular pathology of human glioma. Spectrochim Acta Part A Mol Biomol Spectrosc.

[11] Zhang Y, Xu Y, Liu H, Sun B (2022). Ultrahigh sensitivity nitrogen-doping carbon nanotubes-based metamaterial-free flexible terahertz sensing platform for insecticides detection. Food Chem.

[12] Chen T, Yu L, Tang Z, Li Z, Hu F (2022). Differences in intermolecular interactions between 4-hydroxycoumarin and 7-hydroxycoumarin studied by terahertz spectroscopy and density functional theory. Chem Phys.

[13] Zhan X, Yang S, Huang G, Yang L, Zhang Y, Tian H, Xie F, Lamy de la Chapelle M, Yang X, Fu W (2021). Streptavidin-functionalized terahertz metamaterials for attomolar exosomal microRNA assay in pancreatic cancer based on duplex-specific nuclease-triggered rolling circle amplification. Biosens Bioelectron.

[14] Zhang Q, Li M, Liao T, Cui X (2018). Design of beam deflector, splitters, wave plates and metalens using photonic elements with dielectric metasurface. Opt Commun.

[15] Rouhi K, Rajabalipanah H, Abdolali A (2019). Multi-bit graphene-based bias-encoded metasurfaces for real-time terahertz wavefront shaping: from controllable orbital angular momentum generation toward arbitrary beam tailoring. Carbon.

[16] Zhou Q, Qiu Q, Huang Z (2022). Graphene-based terahertz optoelectronics. Opt Laser Technol.

[17] Hasan M, Arezoomandan S, Condori H, Sensale-Rodriguez B (2016). Graphene terahertz devices for communications applications. Nano Commun Netw.

[18] Li F, Li Y, Tang T, Liao Y, Lu Y, Liu X, Wen Q (2022). Metal-graphene hybrid terahertz metasurfaces based on bound states in the continuum (BIC) and quasi-BIC for dynamic near-field imaging. J Alloys Compd.

[19] Yao H, Yang M, Yan X, Liang L, Sun Z, Yang Q, Wang T, Hu X, Wang Z, Li Z, Wang M, Lv K, Wang Y, Yao J (2022). Patterned graphene and terahertz metasurface-enabled multidimensional ultra-sensitive flexible biosensors and bio-assisted optical modulation amplification. Results Phys.

[20] Rane S, Kothuru A, Jana A, Devi KM, Goel S, Prabhu S, Chowdhury DR (2022). Broadband terahertz characterization of graphene oxide films fabricated on flexible substrates. Opt Mater.

[21] Cheng R, Zhou Y, Wei R, Liu J, Liu H, Zhou X, Cai M, Pan X (2022). Doubling and tripling the absorption peaks of a multi-band graphene terahertz absorber. Diam Relat Mater.

[22] Li S, Xu S, Pan K, Du J, Qiu J (2022). Ultra-thin broadband terahertz absorption and electromagnetic shielding properties of MXene/rGO composite film. Carbon.

[23] Gayduchenko IA, Moskotin MV, Matyushkin YE, Rybin MG, Obraztsova ED, Ryzhii VI, Goltsman GN, Fedorov GE (2018). The detection of sub-terahertz radiation using graphene-layer and graphene-nanoribbon FETs with asymmetric contacts. Mater Today Proc.

[24] Liu C, Wang L, Chen X, Zhou J, Hu W, Wang X, Li J, Huang Z, Zhou W, Tang W, Xu G, Wang S-W, Lu W (2018). Room-temperature photoconduction assisted by hot-carriers in graphene for sub-terahertz detection. Carbon.

[25] Farmani H, Farmani A (2020). Graphene sensing nanostructure for exact graphene layers identification at terahertz frequency. Phys E Low-Dimens Syst Nanostruct.

[26] Enoki T (2011). 2010 Nobel Prize in Physics and research on graphene. Carbon.

[27] Yang Z, Tian J, Yin Z, Cui C, Qian W, Wei F (2019). Carbon nanotube- and graphene-based nanomaterials and applications in high-voltage supercapacitor: a review. Carbon.

[28] Ghosal K, Mondal P, Bera S, Ghosh S (2021). Graphene family nanomaterials—opportunities and challenges in tissue engineering applications. FlatChem.

[29] Hu T, Zhang J, Whyte J, Fu B, Song C, Shang W, Tao P, Deng T (2022). Silicone oil nanofluids dispersed with mesoporous crumpled graphene for medium-temperature direct absorption solar-thermal energy harvesting. Sol Energy Mater Sol Cells.

[30] Kumar R, Singh R, Olabi A-G (2018). Prospect of graphene for use as sensors in miniaturized and biomedical sensing devices☆. Encyclopedia of smart materials.

[31] Fichera L, Li-Destri G, Tuccitto N (2021). Graphene Quantum Dots enable digital communication through biological fluids. Carbon.

[32] Li Q, Tian Z, Zhang X, Xu N, Singh R, Gu J, Lv P, Luo L-B, Zhang S, Han J, Zhang W (2015). Dual control of active graphene–silicon hybrid metamaterial devices. Carbon.

[33] Attariabad A, Pourziad A, Bemani M (2022). A tunable and compact footprint plasmonic metasurface integrated graphene photodetector using modified omega-shaped nanoantennas. Opt Laser Technol.

[34] Nordin NM, Buys YF, Anuar H, Ani MH, Pang MM (2019). Development of conductive polymer composites from PLA/TPU blends filled with graphene nanoplatelets. Mater Today Proc.

[35] Li W, Yang W, Wu M, Zhao M, Lu X (2022). Polydopamine-coated graphene for supercapacitors with improved electrochemical performances and reduced self-discharge. Electrochim Acta.

[36] Pinilla-Sánchez A, Chávez-Angel E, Murcia-López S, Carretero NM, Palardonio SM, Xiao P, Rueda-García D, Torres CMS, Gómez-Romero P, Martorell J, Ros C (2022). Controlling the electrochemical hydrogen generation and storage in graphene oxide by in-situ Raman spectroscopy. Carbon.

[37] Sasikala R, Kandasamy M, Ragavendran V, Suresh S, Sasirekha V, Murugesan S, Sagadevan S, Mayandi J (2022). Perovskite zinc titanate-reduced graphene oxide nanocomposite photoanode for improved photovoltaic performance in dye-sensitized solar cell. Phys B.

[38] Fan C, Jiang W, Yin H, Zhan Y, Wang J (2022). Highly tunable and sensitive plasmon induced transparency modulator with graphene metasurface. Phys E.

[39] Patel SK, Charola S, Jadeja R, Nguyen TK, Dhasarathan V (2021). Wideband graphene-based near-infrared solar absorber using C-shaped rectangular sawtooth metasurface. Phys E.

[40] Chen D, Yang J, Huang J, Bai W, Zhang J, Zhang Z, Xu S, Xie W (2019). The novel graphene metasurfaces based on split-ring resonators for tunable polarization switching and beam steering at terahertz frequencies. Carbon.

[41] Li H, Xing J, Shi Y, Yu S, Zhao T (2023). Performance analysis and optimization of high Q-factor toroidal resonance refractive index sensor based on all-dielectric metasurface. Opt Laser Technol.

[42] Nag A, Simorangkir RBVB, Gawade DR, Nuthalapati S, Buckley JL, O'Flynn B, Altinsoy ME, Mukhopadhyay SC (2022). Graphene-based wearable temperature sensors: a review. Mater Des.

[43] Zhou Y, Wan Y, He M, Li Y, Wu Q, Yao H (2022). Determination of EGFR-overexpressing tumor cells by magnetic gold-decorated graphene oxide nanocomposites based impedance sensor. Anal Biochem.

[44] Rasch F, Postica V, Schütt F, Mishra YK, Nia AS, Lohe MR, Feng X, Adelung R, Lupan O (2020). Highly selective and ultra-low power consumption metal oxide based hydrogen gas sensor employing graphene oxide as molecular sieve. Sens Actuators B Chem.

[45] Rakhshani MR (2021). Wide-angle perfect absorber using a 3D nanorod metasurface as a plasmonic sensor for detecting cancerous cells and its tuning with a graphene layer. Photonics Nanostruct Fundam Appl.

[46] Yang M, Li T, Gao J, Yan X, Liang L, Yao H, Li J, Wei D, Wang M, Zhang T, Ye Y, Song X, Zhang H, Ren Y, Ren X, Yao J (2021). Graphene–polyimide-integrated metasurface for ultrasensitive modulation of higher-order terahertz fano resonances at the Dirac point. Appl Surf Sci.

[47] Wang Z, Wang G, Hu B, Liu W, Huang J, Xiong C, Zhang Y, Liu J, Wang Y (2022). Fast-printed, large-area and low-cost terahertz metasurface using laser-induced graphene. Carbon.

[48] Li Z, Jiao B, Liu W, Hu Q, Li Y, Sun X, Zhao J, Bing P, Zhang H, Zhong K, Yao J (2021). High-efficiency terahertz wave generation combined with optimized cascaded difference frequency generation and optical parametric oscillator. Optik.

[49] Sun L, Zhang H, Dong G, Li P, Zhu Z, Li Y, Guan C, Shi J (2019). Dynamically tunable terahertz anomalous refraction and reflection based on graphene metasurfaces. Opt Commun.

[50] Wang S, Zhao X, Wang S, Li Q, Zhu J, Han L (2020). The investigation of the electromagnetic coupling effect in terahertz toroidal metasurfaces and metamaterials. J Market Res.

[51] Yue Z, Li J, Zheng C, Li J, Chen M, Xu H, Zhang Y, Yao J (2022). Resonance-trapped bound states in the continuum via all-silicon terahertz metasurface. Opt Commun.

[52] Shi Z, Zhang H, Khan K, Cao R, Zhang Y, Ma C, Tareen AK, Jiang Y, Jin M, Zhang H (2022). Two-dimensional materials toward terahertz optoelectronic device applications. J Photochem Photobiol C.

[53] Johnston MB, Joyce HJ (2022). Polarization anisotropy in nanowires: fundamental concepts and progress towards terahertz-band polarization devices. Progress Quantum Electron.

[54] Fateev DV, Mashinsky KV, Sun JD, Popov VV (2019). Enhanced plasmonic rectification of terahertz radiation in spatially periodic graphene structures towards the charge neutrality point. Solid-State Electron.

[55] Song C, Wang J, Zhang B, Qu Z, Jing H, Kang J, Hao J, Duan J (2022). Dual-band/ultra-broadband switchable terahertz metamaterial absorber based on vanadium dioxide and graphene. Opt Commun.

[56] Chen M, Xiao Z (2022). Metal-graphene hybrid terahertz metamaterial based on dynamically switchable electromagnetically induced transparency effect and its sensing performance. Diam Relat Mater.

[57] Zhao Q, Chu C, Xiao X, Chen B (2021). Selectively coupled small Pd nanoparticles on sp2-hybridized domain of graphene-based aerogel with enhanced catalytic activity and stability. Sci Total Environ.

[58] Dong G, Zhang Y, Pan Q, Qiu J (2014). A fantastic graphitic carbon nitride (g-C_3_N_4_) material: electronic structure, photocatalytic and photoelectronic properties. J Photochem Photobiol C.

[59] Cao G, Liu X, Liu W, Li Q, Li X, Wang X (2017). Chemical environment dominated Fermi level pinning of a graphene gas sensor. Carbon.

[60] Çelik O, Saylan Y, Göktürk I, Yılmaz F, Denizli A (2023). A surface plasmon resonance sensor with synthetic receptors decorated on graphene oxide for selective detection of benzylpenicillin. Talanta.

[61] Azeman NH, Bakar MHA, Nazri NAA, Mobarak NN, Khushaini MAA, Aziz THTA, Zain ARM, Bakar AAA (2022). Carboxymethyl chitosan/graphene oxide/silver nanotriangles nanohybrid as the sensing materials for the enhancement of ammonia localized surface plasmon resonance sensor. Opt Laser Technol.

[62] Guo Z, Li J, Weng J, Li J, Chen S, Xu P, Liu W, Wen K, Qin Y (2022). Multiple and tunable plasmon induced transparency with L-shape graphene strips structure at terahertz frequency. Opt Commun.

[63] Alzahrani FA, Sorathiya V (2023). Phase change material and MXene composited refractive index sensor for a wide range of sensing applications at visible and infrared wavelength spectrum. Optik.

[64] Yao CH, Wang ZY, Zhao YM (2021). A leap-frog finite element method for wave propagation of Maxwell–Schrödinger equations with nonlocal effect in metamaterials. Comput Math Appl.

[65] Dash S, Patnaik A, Kaushik BK (2019). Performance enhancement of graphene plasmonic nanoantennas for THz communication. IET Microwaves Antennas Propag.

[66] Menendez GA, Maes B (2017). Time reflection and refraction of graphene plasmons at a temporal discontinuity. Opt Lett.

[67] Maslov A, Bakunov M (2018). Temporal scattering of a graphene plasmon by a rapid carrier density decrease. Optica.

[68] Gong J, Shi X, Lu Y, Hu F, Zong R, Li G (2021). Dynamically tunable triple-band terahertz perfect absorber based on graphene metasurface. Superlattices Microstruct.

[69] Zhou R, Wang C, Huang Y, Huang K, Wang Y, Xu W, Xie L, Ying Y (2021). Label-free terahertz microfluidic biosensor for sensitive DNA detection using graphene-metasurface hybrid structures. Biosens Bioelectron.

[70] Cen W, Lang T, Wang J, Xiao M (2022). High-Q Fano terahertz resonance based on bound states in the continuum in all-dielectric metasurface. Appl Surf Sci.

[71] Zeng Q, Liu W, Lin S, Chen Z, Zeng L, Hu F (2022). Aptamer HB5 modified terahertz metasurface biosensor used for specific detection of HER2. Sens Actuators B Chem.

[72] Gong Y, Hu F, Jiang M, Zhang L, Zou Y, Jiang G, Liu Y (2021). Terahertz binary coder based on graphene metasurface. Carbon.

[73] Li J, Li J, Yang Y, Li J, Zhang Y, Wu L, Zhang Z, Yang M, Zheng C, Li J, Huang J, Li F, Tang T, Dai H, Yao J (2020). Metal-graphene hybrid active chiral metasurfaces for dynamic terahertz wavefront modulation and near field imaging. Carbon.

[74] Islam MS, Sultana J, Biabanifard M, Vafapour Z, Nine MJ, Dinovitser A, Cordeiro CMB, Ng BWH, Abbott D (2020). Tunable localized surface plasmon graphene metasurface for multiband superabsorption and terahertz sensing. Carbon.

[75] Zhang Z, Yan X, Liang L, Wei D, Wang M, Wang Y, Yao J (2018). The novel hybrid metal-graphene metasurfaces for broadband focusing and beam-steering in farfield at the terahertz frequencies. Carbon.

[76] Xie Q, Guo L, Zhang Z, Gao P, Wang M, Xia F, Zhang K, Sun P, Dong L, Yun M (2022). Versatile terahertz graphene metasurface based on plasmon-induced transparency. Appl Surf Sci.

[77] Ghosh R, Aslam M, Kalita H (2022). Graphene derivatives for chemiresistive gas sensors: a review. Mater Today Commun.

[78] Zhang Z, Zhong C, Fan F, Liu G, Chang S (2021). Terahertz polarization and chirality sensing for amino acid solution based on chiral metasurface sensor. Sens Actuators B Chem.

[79] Patel SK, Parmar J, Kosta YP, Ladumor M, Zakaria R, Nguyen TK, Dhasarathan V (2020). Design of graphene metasurface based sensitive infrared biosensor. Sens Actuators A.

[80] Artiles MS, Rout CS, Fisher TS (2011). Graphene-based hybrid materials and devices for biosensing. Adv Drug Deliv Rev.

[81] Ruan B, Guo J, Wu L, Zhu J, You Q, Dai X, Xiang Y (2017). Ultrasensitive terahertz biosensors based on Fano resonance of a graphene/waveguide hybrid structure. Sensors.

[82] Zhang Y, Li T, Zeng B, Zhang H, Lv H, Huang X, Zhang W, Azad AK (2015). A graphene based tunable terahertz sensor with double Fano resonances. Nanoscale.

[83] Tang J, Ye Y, Xu J, Zheng Z, Jin X, Jiang L, Jiang J, Xiang Y (2020). High-sensitivity terahertz refractive index sensor in a multilayered structure with graphene. Nanomaterials.

[84] Nickpay M-R, Danaie M, Shahzadi A (2022). Highly sensitive THz refractive index sensor based on folded split-ring metamaterial graphene resonators. Plasmonics.

[85] Zhang J, Mu N, Liu L, Xie J, Feng H, Yao J, Chen T, Zhu W (2021). Highly sensitive detection of malignant glioma cells using metamaterial-inspired THz biosensor based on electromagnetically induced transparency. Biosens Bioelectron.

[86] Patel SK, Parmar J, Sorathiya V, Zakaria RB, Nguyen TK, Dhasarathan V (2021). Graphene-based plasmonic absorber for biosensing applications using gold split ring resonator metasurfaces. J Lightwave Technol.

[87] Lu Z, Lu N, Xiao Y, Zhang Y, Tang Z, Zhang M (2022). Metal-nanoparticle-supported nanozyme-based colorimetric sensor array for precise identification of proteins and oral bacteria. ACS Appl Mater Interfaces.

[88] Patel SK, Parmar J, Sorathiya V, Nguyen TK, Dhasarathan V (2021). Tunable infrared metamaterial-based biosensor for detection of hemoglobin and urine using phase change material. Sci Rep.

[89] Xu L, Xu J, Liu W, Lin D, Lei J, Zhou B, Shen Y, Deng X (2022). Terahertz metal-graphene hybrid metamaterial for monitoring aggregation of Aβ_16–22_ peptides. Sens Actuators B Chem.

[90] Parmar J, Patel SK (2022). Tunable and highly sensitive graphene-based biosensor with circle/split ring resonator metasurface for sensing hemoglobin/urine biomolecules. Phys B.

[91] Ali MR, Bacchu MS, Das S, Akter S, Rahman MM, Aly MAS, Khan MZH (2023). Label free flexible electrochemical DNA biosensor for selective detection of Shigella flexneri in real food samples. Talanta.

[92] Ye Y, Xie J, Ye Y, Cao X, Zheng H, Xu X, Zhang Q (2018). A label-free electrochemical DNA biosensor based on thionine functionalized reduced graphene oxide. Carbon.

[93] Lee S-H, Choe J-H, Kim C, Bae S, Kim J-S, Park Q-H, Seo M (2020). Graphene assisted terahertz metamaterials for sensitive bio-sensing. Sens Actuators B Chem.

[94] Debia NP, Rodríguez JJP, da Silveira CH, Chaves OA, Iglesias BA, Rodembusch FS, Lüdtke DS (2020). Synthesis and photophysics of benzazole based triazoles with amino acid-derived pendant units. Multiparametr Opt Sens BSA CT-DNA Sol J Mol Liq.

[95] Xu W, Huang Y, Zhou R, Wang Q, Yin J, Kono J, Ping J, Xie L, Ying Y (2020). Metamaterial-free flexible graphene-enabled terahertz sensors for pesticide detection at bio-interface. ACS Appl Mater Interfaces.

[96] Yang K, Li J, Lamy de la Chapelle M, Huang G, Wang Y, Zhang J, Xu D, Yao J, Yang X, Fu W (2021). A terahertz metamaterial biosensor for sensitive detection of microRNAs based on gold-nanoparticles and strand displacement amplification. Biosens Bioelectron.

[97] Meng Q, Ding J, Peng B, Zhang B, Qian S, Su B, Zhang C (2022). Terahertz modulation characteristics of three nanosols under external field control based on microfluidic chip. iScience.

[98] Patel SK, Surve J, Parmar J (2022). Detection of cancer with graphene metasurface-based highly efficient sensors. Diam Relat Mater.

[99] Zhao X, Yi J, Zhu L, Wang J, Lin M, Chen X, Burokur SN (2021). Adsorption of graphene-based metamaterials and its application in detection of heavy metal ions. Opt Mater Express.

[100] Ding J, Arigong B, Ren H, Shao J, Zhou M, Lin Y, Zhang H (2015). Dynamically tunable Fano metamaterials through the coupling of graphene grating and square closed ring resonator. Plasmonics.

[101] Piccinini E, Allegretto JA, Scotto J, Cantillo AL, Fenoy GE, Marmisollé W, Azzaroni O (2021). Surface engineering of graphene through heterobifunctional supramolecular-covalent scaffolds for rapid COVID-19 biomarker detection. ACS Appl Mater Interfaces.

[102] Zangeneh-Nejad F, Safian R (2016). A graphene-based THz ring resonator for label-free sensing. IEEE Sens J.

[103] Amin M, Siddiqui O, Abutarboush H, Farhat M, Ramzan R (2021). A THz graphene metasurface for polarization selective virus sensing. Carbon.

[104] Zhang D, Sun D, Wen Q, Wen T, Kolodzey J, Zhang H (2016). Tuning the optical modulation of wideband terahertz waves by the gate voltage of graphene field effect transistors. Compos B Eng.

[105] Wang Y, Cui Z, Zhang X, Zhang X, Zhu Y, Chen S, Hu H (2020). Excitation of surface plasmon resonance on multiwalled carbon nanotube metasurfaces for pesticide sensors. ACS Appl Mater Interfaces.

[106] Deng X, Shen Y, Liu B, Song Z, He X, Zhang Q, Ling D, Liu D, Wei D (2022). Terahertz metamaterial sensor for sensitive detection of citrate salt solutions. Biosensors (Basel).

[107] Lin S, Xu X, Hu F, Chen Z, Wang Y, Zhang L, Peng Z, Li D, Zeng L, Chen Y, Wang Z (2021). Using antibody modified terahertz metamaterial biosensor to detect concentration of carcinoembryonic antigen. IEEE J Sel Top Quantum Electron.

[108] Wang G, Zhu F, Lang T, Liu J, Hong Z, Qin J (2021). All-metal terahertz metamaterial biosensor for protein detection. Nanoscale Res Lett.

[109] Zhang Z, Yang G, Fan F, Zhong C, Yuan Y, Zhang X, Chang S (2021). Terahertz circular dichroism sensing of living cancer cells based on microstructure sensor. Anal Chim Acta.

[110] Yang X, Yang K, Zhao X, Lin Z, Liu Z, Luo S, Zhang Y, Wang Y, Fu W (2017). Terahertz spectroscopy for the isothermal detection of bacterial DNA by magnetic bead-based rolling circle amplification. Analyst.

[111] Iwanaga M (2020). All-dielectric metasurface fluorescence biosensors for high-sensitivity antibody/antigen detection. ACS Nano.

[112] Ahmed R, Guimaraes CF, Wang J, Soto F, Karim AH, Zhang Z, Reis RL, Akin D, Paulmurugan R, Demirci U (2022). Large-scale functionalized metasurface-based SARS-CoV-2 detection and quantification. ACS Nano.

[113] Ahmadivand A, Gerislioglu B, Ramezani Z, Kaushik A, Manickam P, Ghoreishi SA (2021). Functionalized terahertz plasmonic metasensors: femtomolar-level detection of SARS-CoV-2 spike proteins. Biosens Bioelectron.

[114] Ma B, Ouyang A, Zhong J, Belov PA, Sinha RK, Qian W, Ghosh P, Li Q (2021). All-dielectric metasurface for sensing microcystin-LR. Electronics.

[115] Wang C, Huang Y, Zhou R, Xie L, Ying Y (2020). Rapid analysis of a doxycycline hydrochloride solution by metallic mesh device-based reflection terahertz spectroscopy. Opt Express.

[116] John-Herpin A, Kavungal D, von Mucke L, Altug H (2021). Infrared metasurface augmented by deep learning for monitoring dynamics between all major classes of biomolecules. Adv Mater.

